# Contemporary Classification and Diagnostic Pathways for Common Gingival and Alveolar Ridge Lesions

**DOI:** 10.1155/ijod/7197720

**Published:** 2026-10-12

**Authors:** Hamed Mortazavi, Aida Kheiri, Shaghayegh Khoshgou, Ehsan Chegeni, Hamidreza Eghbalikhosro, Amirsalar Mofid, Zahra Karami

**Affiliations:** ^1^ Department of Oral Medicine School of Dentistry Shahid Beheshti University of Medical Sciences Tehran Iran sbmu.ac.ir; ^2^ Department of Periodontics School of Dentistry Shahid Beheshti University of Medical Sciences Tehran Iran sbmu.ac.ir; ^3^ Dental Research Center Research Institute of Dental Sciences Shahid Beheshti University of Medical Sciences Tehran Iran sbmu.ac.ir

**Keywords:** alveolar process, decision trees, gingiva, mouth disease, oral ulcer, periodontium

## Abstract

Oral lesions can be observed in different locations in the oral cavity, including periodontal supporting structures, gingiva, and alveolar ridge. Ranging from benign to potentially malignant lesions, these lesions can be described from various perspectives, including duration, size, origin, number, and clinical characteristics. Early diagnosis of these lesions can highly affect treatment procedures and outcomes, while preserving tooth‐supporting tissues. This review aimed to introduce a decision tree for gingival and alveolar ridge lesions. According to the search strategy, a thorough electronic search was conducted in Web of Science, MEDLINE/PubMed, Google Scholar, EMBASE, and Scopus to identify English‐language studies published from January 2000 to June 2026. All clinical studies, including case reports and series, clinical trials and cohorts, and reviews, were considered eligible for inclusion. Initial search led to 6472 findings. Following screening and full‐text assessment, 129 studies were finally included. According to clinical manifestations, four main groups of gingival and alveolar ridge lesions, named ulcers and erosions, exophytic lesions, white lesions, and pigmented lesions, were introduced. Based on the clinical characteristics of lesions in each group, certain entities were identified for each category. This review presented a clinically based decision tree to enhance the diagnostic accuracy of gingival and alveolar ridge lesions. Applying this decision tree in clinical practice can help with precise diagnosis and appropriate treatment selection, thereby further improving therapeutic outcomes and preserving the integrity of periodontal structures around teeth.

## 1. Introduction

A wide variety of mucosal lesions can occur in the oral cavity with different etiologies and risk factors. They mainly range from benign reactive lesions to more serious carcinomatous ones. Thus, accurate diagnosis of these lesions is of great importance for establishing an appropriate treatment approach with minimal delay [1, 2].

An oral lesion can be described using different criteria, including size, number, origin, color, consistency, surface texture, clinical appearance, duration, and so forth. Generally, oral lesions are divided into four subgroups: exophytic lesions, ulcerations, pigmentation, and white lesions [3].

The periodontium structure comprise of four main components, from which the gingiva and alveolar ridge can present as involved sites in many oral pathologies. Gingiva is a common site for not only infections and traumas but also more important conditions like squamous cell carcinoma (SCC) and autoimmune bullous disease. It also represents one of the locations with patient discomfort and further impact on quality of life [4–6]. Therefore, a logical diagnostic pattern, such as decision trees organized into categories, can be proposed using definitive features to identify the main lesion etiologies and assess the possibility of malignancy [2]. Hence, the present narrative review aimed to introduce an updated decision tree focusing on two important parts of periodontal tissues, the gingiva and the alveolar ridge, based on clinical manifestations.

## 2. Methodology

In order to find relative articles for the present narrative review, this narrative review, a thorough search was performed across the main search engines, including PubMed/MEDLINE, Web of Science, Scopus, EMBASE, and Google Scholar, as well as textbooks, to identify relevant English‐language articles from January 2000 until June 2026. MESH keywords entailing “oral lesions,” “oral disease,” “[oral OR gingival OR alveolar] AND lesion” were used. All types of studies, including case reports, case series, original studies (randomized clinical trials, nonrandomized clinical trials, and prospective or retrospective cohorts), and reviews of clinical studies, were primarily considered appropriate. Editorials and letters to the editor were not considered eligible.

Out of 1336 full‐text assessments, finally, 129 studies were considered eligible for our study. 1207 articles were excluded due to lack of full texts, being written in languages other than English, focusing on histopathologic features of lesions rather than clinical aspects, or containing overlapped material. After gathering clinical‐related data like common location, number, color, surface texture, and duration, gingival and alveolar lesions were categorized into four main groups: ulcerations and erosions, exophytic lesions, white lesions, and pigmented lesions. Based on the duration and number of ulcerative lesions, they were further classified into acute, chronic, and recurrent groups, as well as single‐ or multiple‐subgroup groups. Concerning surface characteristics (smooth/rough), exophytic lesions were assigned to six subgroups. White lesions were defined in two subgroups: scrubbable and unscrubbable. This classification was based on the possibility of scraping the white lesion off the underlying mucosa with a sterile gauze. Pigmented lesions, the 4^th^ group, were also subdivided into four parts. Figures 1–3 represent the final decision tree for gingival and alveolar ridge lesions. Table 1 summarizes the lesions, common features, and their clinical characteristics.

**Figure 1:**
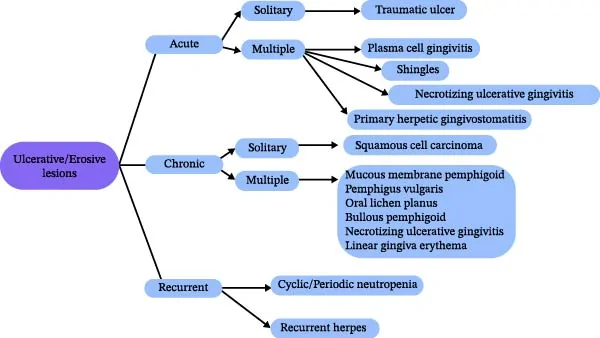
Decision tree for common ulcerative and erosive lesions on gingiva and alveolar ridge.

**Figure 2:**
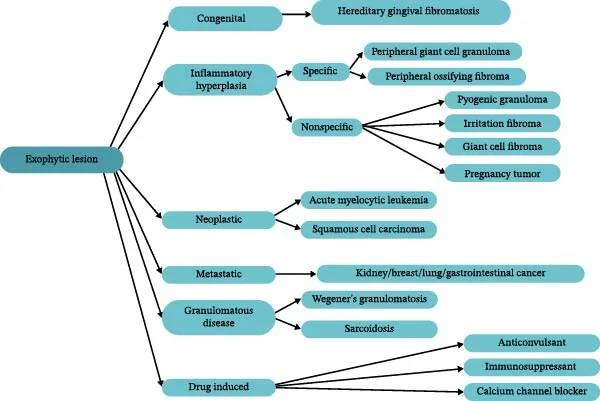
Decision tree for common exophytic lesions on gingiva and alveolar ridge.

**Figure 3:**
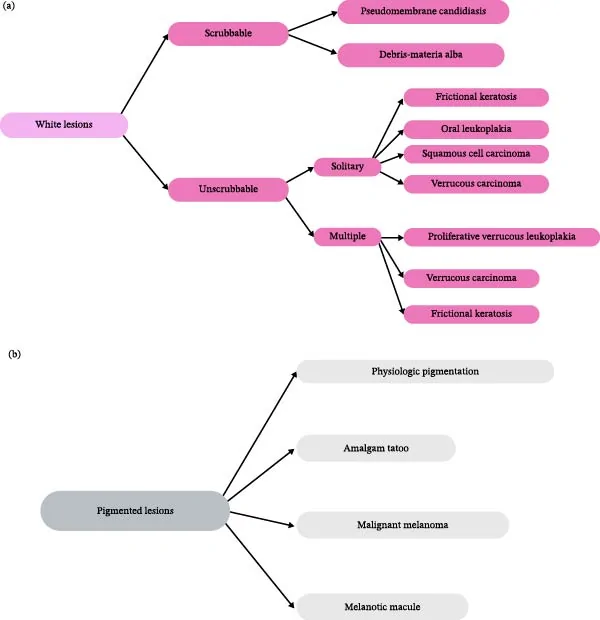
(a) Decision tree for common white lesions on gingiva and alveolar ridge. (b) Decision tree for common pigmented lesions on gingiva and alveolar ridge.

**Table 1:** Characteristics of gingival and alveolar ridge lesions.

Lesion	Gender predominance	Age predilection	Common location	Clinical manifestation	Treatment options
Gingival/alveolar ridge ulceration/erosions					

Traumatic ulcer	Male (reported ratio: 2.7:1)	Usually in young patients	Mostly buccal mucosa and lateral border of tongue, gingiva.	Solitary slightly symmetrical/asymmetrical ulceration with raised, reddish borders and a scrubbable yellowish‐white necrotic pseudo membrane.	Painless healing within 3–10 days after elimination of injury cause.
Plasma cell gingivitis	Female (reported ratio: 2.6:1)	Wide age range (9–64 years of age)	Anterior maxillary gingiva	Multiple erythematous gingiva with a velvety texture and loss of stippling.	‐Usually self‐limiting. ‐Pain control and anti‐inflammatory agents for better healing period.
Shingles	NS	>50 years old	Hard palate, buccal gingiva	Clustered or zosteriform pattern, unilateral painful, ulcers of 1–5 mm in diameter with scalloped borders which usually heal within 10–14 days.	‐Acyclovir, valacyclovir, or famciclovir is effective when started within 72 h of disease onset. ‐Pain control, supportive care, and hydration.
Necrotizing ulcerative gingivitis	NS	Usually young people (18–20 years of age)	Marginal and papillary gingiva	Multiple punched‐out/crater like ulceration + necrosis of interdental papillae, sore gums, bleeding gums.	Mechanical removal of tartar with CHX (BID) and systemic (amoxicillin 250 mg and metronidazole 250 mg,TDS for 7 days) + delivery of antimicrobial agents.
Primary herpetic gingivostomatitis	NS	Two age peaks: 6 months and 5 years	Gingiva, labial mucosa and palate	Multiple large, deep, necrotic ulcerations surrounded by a yellow, circinate hypertrophic border, presenting atypical features in immunocompromised patients.	Spontaneous healing after 5–7 days without no scar formation.
Squamous cell carcinoma	Male	>40 years	Posterior lateral borders of the tongue, floor of the mouth, soft palate, gingiva, buccal mucosa, and hard palate.	Solitary ulceration with rolled and indurated borders, with a velvety base.	Therapeutic modalities include surgery, radiotherapy, and chemotherapy or even combination different methods.
Mucous membrane/ cicatricial pemphigoid	Female (reported ratio: 2:1)	50–70 years	Gingiva, buccal mucosa and palate	Multiple erosions with a nonspecific appearance, representing as a desquamative gingivitis.	Topical or systemic corticosteroids application for oral involvement.
*Pemphigus vulgaris*	Almost equally in both genders	~50 years	Buccal mucosa, gingiva, and palate	Multiple map‐like erosions and shallow ulcers with peripheral extension, desquamative gingivitis.	High doses of systemic corticosteroids, usually given in dosages of 1to2 mg/kg/d.
Oral lichen planus	Female (reported ratio: 2:1)	50–55 years	Buccal mucosa, tongue, gingiva, palate, and vermilion border	Multiple/reticular (characterized by Wickham’s striae), popular, and plaque‐like lesions are usually asymptomatic. atrophic, erosive/ulcerative, bullous lesions are painful. Desquamative gingivitis	Topical or systemic corticosteroids recommended for oral lichen planus.
Bullous pemphigoid	NS	>60 years old	Attached gingiva, soft palate, buccal mucosa, and floor of the mouth.	Discrete vesicle formation, multiple desquamative gingivitis, generally self‐limiting, early remission.	‐Topical clobetasol or betamethasone for localized lesions. ‐In more extensive disease, use of systemic corticosteroids alone or in combination with immunosuppressive drugs is recommended.
Linear gingiva erythema	Female	6^th^ and 7^th^ decade	Anterior gingiva	Multiple petechiae‐like or diffuse red lesions erythematous linear band, Bleeding.	‐Oral lesions managed with the use of topical steroids. ‐Dapsone therapy for more severe cases is recommended. ‐Resistant cases may require systemic corticosteroids.
Cyclic/periodic neutropenia	NS	Childhood	Any part of oral mucosa exposed to trauma.	Multiple painful, irregular, and deep oral ulcers, often covered with a yellowish, pseudomembranous layer (aphthous like). Other manifestations include gingivitis, & periodontitis, often accompanied by other systemic symptoms such as fever and malaise.	Administration of granulocyte colony‐stimulating factor (G‐CSF).
Recurrent herpes	NS	Adult	Vermilion/ intra oral (keratinized mucosa: hard palate or attached gingiva).	Multiple/ Cluster of vesicles on keratinized mucosa accompanied by underlying erythema.	‐Symptomatic treatment by using ice or lanolin. ‐Applying acyclovir ointment 5% every 2 hours since the prodromal phase until the lesions subside.

Gingival/alveolar ridge exophytic lesions					

Hereditary gingival fibromatosis	NS	Childhood/adolescence	Free and attached gingiva (often anterior); maxillary tuberosities; retromolar pads.	‐Smooth texture with broad base and firm/fibrotic consistency. ‐Pink; generalized overgrowth. ‐Slowly progressive enlargement; may impair mastication or speech.	Surgical excision.
Peripheral giant cell granuloma	Female	Adults (5^th^–6^th^ decade)	Gingiva/alveolar ridge (mandible > maxilla).	Nodular texture with soft to firm consistency, red–purple to bluish‐brown color; usually < 2 cm. “Dumb‐bell” shape; may cause tooth displacement.	Complete surgical excision.
Peripheral ossifying fibroma	Female	10–19 years	Interdental papilla (anterior maxilla predilection).	Smooth surface, sessile or pedunculated, firm consistency and red–pink color; usually < 1.5 cm. Possible tooth mobility or underlying bone resorption.	Surgical excision involving the underlying periosteum.
Pyogenic granuloma	Female (especially pregnant women)	No age predilection	Gingiva/alveolar ridge	Smooth or lobulated; often ulcerated with sessile or pedunculated base and soft consistency. Bright red to purplish color with rapid growth. Bleeds easily on manipulation.	Conservative surgical excision.
Irritation fibroma (inflammatory fibrous hyperplasia)	Female	5^th^–6^th^ decade	Gingiva, buccal mucosa along occlusal line, interdental papilla.	Smooth texture, sessile or pedunculated with firm consistency. Pink; usually small and nodular. Painless, slow‐growing.	Surgical excision
Giant cell fibroma	Female	3^rd^ decade	Gingiva (mandible > maxilla).	Papillary or granular surface, sessile or pedunculated with firm consistency. Pink; typically < 1 cm.	Surgical excision.
Pregnancy tumor	Pregnant women (female)	Gestational age	Gingiva.	Smooth or lobulated surface with soft consistency. Sessile or pedunculated, Bright red; may become quite large. Rapid enlargement; profuse bleeding; often asymptomatic systemically.	Conservative surgical excision.
Acute myelocytic leukemia	NS	>50 years old	Gingiva	‐Swelling and reddening of the gingival tissues. ‐Advanced cases: accompanied by bone loss and tooth mobility.	Chemotherapy
Metastatic	Male	6^th^ and 7^th^ decade	Gingiva	Swelling, pain, bleeding	Local resection, chemotherapy, radiation therapy or combination of them.
Wegener’s granulomatosis	NS	45–60 years	Gingiva, buccal/labial mucosa	Irregular surface, nodular shape, red color that bleeds easily + strawberry gingivitis + chronic oral ulceration.	Systemic corticosteroids
Sarcoidosis	Female	20–40 years old	Gingiva, buccal mucosa, lip, tongue, palate, salivary gland.	Xerostomia, cobble stone swelling of gingiva.	Systemic corticosteroids + steroid‐sparing agents + surgical excision.
Drug‐induced gingival overgrowth	Male	Younger age	Gingiva (anterior of jaws).	Lobulated Early: pink Advance: reddish‐bluish.	Medical consultation + surgical excision.

Gingival/alveolar ridge white lesions					

Pseudomembranous candidiasis	Female	Infants/elderly	Gingiva, buccal, tongue, palate	Scrubbable. Creamy white plaques, papules, or patches.	Administration of antifungals
Debris‐materia alba	NS	NS	Oral cavity	Scrubbable. Soft, sticky, yellowish, or grayish‐white deposit	No treatment needed
Frictional keratosis	NS	None	Gingiva, linea alba, cheek, lip and tongue	Unscrubbable. Solitary/multiple white plaque Rough and threadlike surface.	Spontaneous healing 2 weeks after irritant removal.
Oral leukoplakia	Male	>50 years	Gingiva, mucosa of the buccal, vermilion of the lip	Unscrubbable White spots and plaque, potentially malignant lesion	Elimination of predisposingfactors, use of β‐carotene, lycopene, ascorbicacid,‐Tocopherol (VitaminE), topical Vitamin A, topical bleomycin, cold‐knife surgical excision, laser surgery.
Squamous cell carcinoma	Male	>65 years	Gingiva, floor of the mouth and borders of the tongue.	Unscrubbable. Red, white, or a combination. Granular, rough, papillary, or scaly, appear as irregular masses or ulcers with rolled margins.	Chemoradiation therapy with or without surgery.
Verrucous carcinoma	Male	Elderly	Vestibule of the mandible, mucosa of the buccal, tongue, gingiva, hard palate.	Unscrubbable. Solitary/multiple thick white plaque with well‐defined borders and papillary or wart‐like surface.	Surgery with or without radiotherapy.
Proliferative verrucous leukoplakia	Female	>5^th^ decade	Mostly gingiva, other oral mucosa	Uncurbbable Small and well‐defined white plaques or plaques appearing as focal and homogeneous keratotic lesions	Surgery, carbon dioxide laser ablation, topical photodynamic therapy, oral retinoids, topical bleomycin solution, β‐carotene, radiation and chemotherapy

Gingival/alveolar ridge pigmented lesions					

Physiologic pigmentation	NS	NS	Gingiva	Asymmetric shape, solitary /multiple, with well‐defined borders.	Laser application for cases with esthetic demands
Amalgam tattoo	NS	NS	Margin of gingiva & alveolar mucosa	Asymmetric sharp, bluish grey macule or plaque with ill‐defined borders.	In esthetic area: surgical excision and gingival grafting.
Malignant melanoma	No specific gender	6^th^ decade	Hardpalate and maxillary gingiva.	‐Black–brown patch, macule, or nodular lesion with different shades of gray, red, purple, or areas of depigmentation. ‐Rapid progression. ‐Ranging from benign appearing lesions to more malignant‐appearing lesions with irregular borders or an exophytic surface proliferation.	Surgery with or without chemoradiation (low survival rate).
Melanotic macule	Female	43 years	Gingiva, lower lip	Symmetric shape, brown‐to‐black/6–10 mm, solitary, small, sharply demarcated, and uniform in color.	Biopsy needed for lesions > 0.5 cm. No further treatment is needed.

*Note:* BID, twice a day; TDS, three times a day; Yrs, years old.

Abbreviations: CHX, chlorohexidine; Cm, centimeter(s); F, female; M, male; Mm, millimeter(s); NS, not specified.

### 2.1. Ulcerative/Erosive Lesions

#### 2.1.1. Acute Solitary Ulcerative/Erosive Lesion

An acute single ulceration is defined as a solitary ulcerative lesion lasting less than 2 weeks [7]. The following section describes the sole entity in this group (Figure 1).

##### 2.1.1.1. Traumatic Ulcer

Mechanical, thermal, electrical, or chemical factors can cause trauma‐induced injuries to the oral mucosa and gingiva [7, 8]. Traumatic ulcers appear in various forms, including aphthous, map‐like, and crater‐like appearances, with both acute and chronic presentations [9]. They can be self‐inflicted, iatrogenic, or accidental [7, 8]. Traumatic ulcers in the oral cavity are most frequently diagnosed on the buccal mucosa (42%), followed by the tongue (25%), and the lower lip (9%) [7]. Thermal factors, toxic chemical products, and iatrogenic misuse may also result in sloughing or ulceration of the gingiva [8, 10]. Inappropriate tooth brushing technique can manifest as shallow gingival lacerations and, in severe cases, lead to gingival recession [11]. Gingival ulceration can also be caused by an inappropriate dental flossing technique, with a major effect on the tips of the interdental papillae [8]. Based on the etiology, traumatic ulcers frequently appear as single, symmetrical, or asymmetrical lesions with nonspecific clinical manifestations, including slightly elevated red borders and a yellow‐to‐white necrotic pseudomembrane. These lesions usually do not require treatment, and, upon removal of the etiologic factor, they can resolve within 10 days [7, 12].

#### 2.1.2. Acute Multiple Ulcerative/Erosive Lesions

The definition of acute multiple ulcerations or erosions is several ulcerative lesions that take more than 2 weeks to heal [7]. The following section describes four entities in this group.

##### 2.1.2.1. Plasma Cell Gingivitis (PCG)

PCG is a nonplaque‐induced gingival condition frequently appearing on the anterior maxillary gingiva. While the exact pathologic mechanism of this lesion remains unknown, it occurs a few days after exposure to an allergen, presenting as erythematous gingiva with a velvety texture and loss of stippling [7, 8, 13]. Common clinical manifestation consist of ulceration, sloughing, and desquamation [7]. Bleeding on provocation and gingival enlargement may also be observed signs in some patients. Histopathologically, it shows polyclonal proliferation of plasma cells in the lamina propria. Because the etiology of this disease is not fully understood, no standard protocol exists, and treatments are aimed at relieving the symptoms [8, 13].

##### 2.1.2.2. Shingles

Herpes zoster infection (HZI), or Shingles, is an uncommon viral infection caused by reactivation of the varicella zoster virus (VZV), which remains latent in dorsal root/cranial nerve ganglia in 1/3 of affected patients [7, 14]. Evidence has shown that some of the contributing factors to VZV reactivation include aging, stress, trauma from dental procedures, malignancy, radiation therapy, and immunodeficiency conditions, or it may even occur spontaneously ^7^. The incidence rate of HZI has been reported to be 1.5–3/1000 cases, with a tendency to increase in patients over 75 years old [15, 16]. Thoracic and lumbar dermatomes are the main affected areas, followed by trigeminal nerve involvement with pain mimicking a toothache [7, 17]. By affecting mandibular and maxillary branches of the trigeminal nerve, skin manifestation may be associated with unilateral oral ulcers encompassing gingival lesions [8, 17]. Intraoral ulcers usually occur over a period of 10–14 days. The disease progression is self‐limiting, and management of oral symptoms is based on pain management, supportive care, and adequate water intake. In the treatment of HZI, medications such as acyclovir, valacyclovir, or famciclovir can be effective only if started within 72 h of illness onset [18, 19]. However, the risk of postherpetic neuralgia (PHN) is estimated between 5% and 30%, and 1/3 of patients with PHN experience chronic pain for even more than 1 year [20].

##### 2.1.2.3. Necrotizing Ulcerative Gingivitis (NUG)

NUG is a severe necrotic condition of the gingiva that results from bacterial infection in patients with specific predisposing factors (psychological stress, poor oral hygiene, malnutrition, and compromised immune status). It is characterized by tissue necrosis on marginal and papillary gingiva and ulcers with a “punched‐out” appearance, severe pain, and gingival bleeding [7, 8]. This disease mostly commences with a localized lesion, but it can laterally spread from the papilla to the gingival margin, both buccally and lingually [21]. As long as the lesion is confined to the gingiva, it is called NUG [22], but in the presence of attachment loss, the diagnosis will be ‘’necrotizing ulcerative periodontitis” (NUP) [23]. Lesions that extend beyond the mucogingival junction by more than 1 cm from the gingival margin are called ‘’necrotizing stomatitis” (NS) [24]. NUG usually affects young people between the ages of 18 and 20, with the whole reported prevalence to be 0.5% to 11% in the population. Three essential symptoms in the diagnosis of NUG are painful gingiva, gingival bleeding, and, most importantly, ulceration and necrosis of the interdental papillae. Treatment includes mechanical removal of the calculus and the use of local antibacterial agents and systemic medications. Complete resolution of the disease occurs after removing local and systemic/environmental conditions [7].

##### 2.1.2.4. Primary Herpetic Gingivostomatitis (PHGS)

Herpes viruses comprise eight subgroups of double‐stranded DNA viruses that can cause human infections [25]. Herpes simplex viruses (HSV‐1 and HSV‐2) are the main two sources for recurrent infections. HSV‐1 primarily causes asymptomatic infection in the orofacial area, with only 1/3 of patients with PHGS showing clinical manifestations [25]. Fever, malaise, myalgia, nausea, cervical lymphadenitis, and headache are among prodromal signs, while the presence of vesicles in cluster morphology, painful erosive lesions, and pseudomembranes are common oral manifestations. The most involved sites in the oral cavity include the gingiva and labial mucosa. Most PHGS cases heal within 10–14 days without scarring; however, periodic viral reactivation triggered by certain factors can lead to recurrent lesions [26]. Hence, severe cases may require intervention to decrease the patients’ discomfort. Systemic antivirals, including acyclovir and its derivatives, are the main options for reducing symptom duration and severity. The initial step in PHGS management includes topical treatments for orolabial lesions, especially when used in the early phase of disease activation. In addition, adjunctive supportive care, such as hydration, can help with pain management [25, 27] (Figure 4).

**Figure 4:**
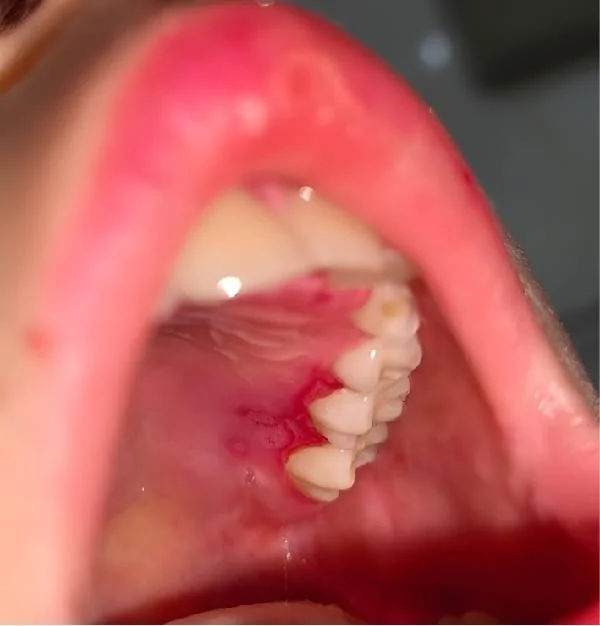
Primary herpetic gingivostomatitis on the palate.

#### 2.1.3. Chronic Solitary Erosive/Ulcerative Lesion

Chronic single ulceration is defined as a sole ulceration that remains in the oral cavity for more than 2 weeks [7]. The only entity in this group is SCC, which is described below.

##### 2.1.3.1. SCC

SCC accounts for more than 90% of all oral malignancies [28]. Clinical manifestation includes a reddish, white, red–white, exophytic, or ulcerative lesion [7]. The classic form of ulcerative SCC is a solitary crater‐like lesion with rolled and indurated borders and a velvety base. However, in rare cases, it may appear as multifocal lesions [29]. The most affected areas by oral SCC are the posterior lateral borders of the tongue, the oral floor, the soft palate, the gingiva, the buccal mucosa, and the hard palate, in that order [2]. SCC of the gingiva accounts for ~20% of oral carcinomas and occurs most commonly in the edentulous area of the posterior mandible [8]. Men older than 40 years are at greater risk for this malignancy [30]. Tobacco and alcohol use, solar exposure, viruses, and oral premalignant lesions may be associated with the etiology of SCC [10]. Early diagnosis of SCC plays an important role not only in the treatment but also in the prognosis. Treatment modalities of SCC range from wide surgical excision, neck dissection, radiation therapy, chemotherapy, or a combination of them [7, 10].

#### 2.1.4. Chronic Multiple Erosive/Ulcerative Lesions

These lesions, including six entities, are known to be more than a single lesion and to last for more than 14 days [7].

##### 2.1.4.1. Mucous Membrane Pemphigoid (MMP)

MMP, also known as cicatricial pemphigoid, is a common autoimmune subepithelial vesiculobullous disease that predominantly involves the oral (85% of cases) and ocular (64% of cases) mucosa, with a recently reported incidence of 1–2 case per million [7, 31–33]. Oral ulcers of MMP manifest as multiple erosions with a nonspecific appearance [7]. These erosions result from autoreactive antibodies to basement‐membrane zone proteins [33]. The gingiva is the most affected site in the oral cavity, followed by the buccal and palatal mucosa [7, 10]. Gingival lesions involve variable degrees of erythema and edema, enlarged interdental papillae, and blistering. Though the presence of blisters in the oral cavity is highly diagnostic for MMP, desquamative gingivitis may be the only sign of the disease [7, 31, 33]. It is mainly observed in patients in the 5^th^ and 6^th^ decades of life, with a female predominance [34]. The management of MMP is performed by topical and systemic corticosteroids for severe cases [7, 35, 36] (Figure 5).

**Figure 5:**
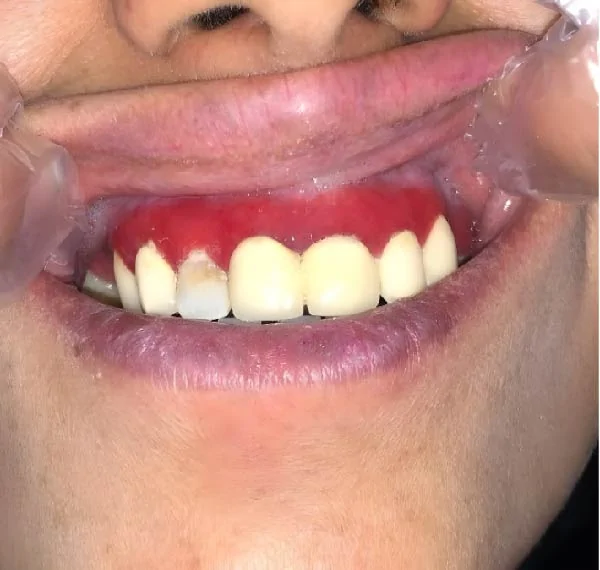
Desquamative gingivitis in a patient with mucous membrane pemphigoid.

##### 2.1.4.2. Pemphigus Vulgaris (PV)

PV is a rare, chronic, immune‐mediated vesiculobullous disease of skin and mucous membranes, with the reported global incidence of 0.1–3.2/100.000 population [32, 33]. This lesion occurs as a result of acantholysis with suprabasilar separation and blister formation [8, 31, 37]. This acantholysis results from the destruction of cell–cell adhesion between keratinocytes due to the production of autoantibodies against desmogleins (Dsg) 1 and 3, which form desmosomes [38]. PV happens equally in both genders with a mean age of 40–60 years [10, 31, 32]. Oral lesions, hallmarks of PV, represent symptoms in almost 50%–90% of cases and mainly affect the buccal mucosa, gingiva, and palate [7, 32, 33, 38, 39]. Lesions of the oral cavity begin as intraepithelial bullae arising on a noninflamed base with quick rupture [7, 8]. Therefore, in most cases, the clinician encounters erosions and shallow ulcers with considerable pain and time to heal. Oral lesions usually occur before skin lesions [7]. The definitive diagnosis is determined by clinical manifestations and histopathologic examination [7, 31]. High doses of systemic corticosteroids remain the gold standard treatment approach for PV. In the case of early diagnosis, reduced doses of medication for short durations can be beneficial in managing the disease [7].

##### 2.1.4.3. Oral Lichen Planus (OLP)

OLP is a chronic, mucocutaneous, immune‐mediated disease that affects T‐cells [7, 10]. With a global prevalence of 1%, this disease typically occurs in individuals aged ~50–55 years [7, 33, 40, 41]. Frequently appearing bilaterally, OLP exists in sites with risk for trauma, like the buccal mucosa, and can happen solely or in conjunction with nonoral involvement [10, 40]. This condition may appear in the gingiva in ~35% of cases [33]. There are different subtypes of OLP with a wide range of clinical manifestations, including reticular, erosive/ulcerative/atrophic, popular/plaque‐like, and bullous. The first three mentioned subtypes are more common than the others. Reticular lesions appear as a keratotic lace‐like pattern known as Wickham’s striae [7, 42]. Keratotic alterations along with erythematous mucosa are observed in the atrophic form [7]. In addition to reticular or erosive patterns, bullous OLP presents with vesiculobullous lesions following vesicle rupture [2, 7]. In erosive/ulcerative OLP, covered ulcerations with pseudomembranes, along with keratosis and erythema, are one of the main observed characteristics [7]. The plaque‐like OLP is a white, homogeneous, mildly raised lesion that mimics leukoplakia. Unlike reticular, plaque‐like, and papular lesions, atrophic, bullous, erosive, and ulcerative forms exhibit varying degrees of discomfort [7, 42]. In addition to the variations of OLP presentations, it is important to note that in 10% of cases, manifestations solely appear on the gingiva, predicting possible involvement of other mucous membranes [33]. Based on disease severity, topical and systemic corticosteroids are recommended in the palliative treatment of OLP [2, 7, 10].

##### 2.1.4.4. Bullous Pemphigoid (BP)

BP is the most prevalent immune‐mediated cutaneous blistering disease, accounting for 80% of cases [7, 31]. In BP, autoantibodies are produced against the self‐antigens BP180 and BP230, which are located in the basement membrane [42]. BP affects both genders equally and occurs mainly in elderly patients with a peak incidence in the 80^th^ decade of life [31]. Mucosal involvement is not common in this disease. Additionally, oral manifestations are observed in only 16.6% of patients. BP’s clinical presentations are similar to those of PV but with less extension and fewer symptoms [7, 43]. The involvement of attached gingiva, called desquamative gingivitis, is observed frequently and may be the only affected intraoral site [7, 31]. However, other areas, such as the soft palate, buccal mucosa, and oral cavity floor, may also be involved [31]. Although the oral lesions of BP are clinically indistinguishable from those of PV or MMP, BP lesions tend to remit earlier [7, 43]. The incidence of BP has been associated with genetic predisposition, malignancies, or certain medications [31, 44]. BP is generally a self‐limiting lesion, and its treatment and prognosis depend on its severity [7, 31]. Topical clobetasol or betamethasone can be prescribed to control localized lesions [7]. However, in extensive cases, systemic corticosteroids and immunosuppressive drugs are recommended [7, 31, 43].

##### 2.1.4.5. NUG

This lesion can present as solitary or multiple lesions. Detailed information regarding its characteristics is given in the previous section.

##### 2.1.4.6. Linear Gingival Erythema (LGE)

LGE is the most common lesion of HIV‐related periodontal manifestation with candidiasis infection source in HIV‐positive patients [44–48]. With considerable prevalence from 0% to 48% [49], it usually manifests as an erythematous linear band extending 2–3 mm from the free gingival margin. It can also show petechiae‐like or diffuse red lesions on the attached gingiva and oral mucosa with signs of bleeding [49, 50].

#### 2.1.5. Recurrent Ulcers

Recurrent ulcers are defined as lesions with similar episodes and intermittent healing [7]. Details of two lesions in this category are described below.

##### 2.1.5.1. Cyclic/Periodic Neutropenia

Cyclic neutropenia, also known as periodic neutropenia, is a rare disorder that significantly affects oral health and is caused by a periodic reduction in the quantity of neutrophils [51]. This condition is characterized by a range of oral manifestations, often recognized as early signs of neutropenic status [51, 52], and the first signs appear in childhood [53].

The most common oral manifestations include oral ulcers, gingivitis, and periodontitis, which are often accompanied by systemic symptoms such as fever and malaise [54]. Oral ulcers may be painful, have a deep and irregular shape, and are often covered with a yellowish pseudomembranous layer. These ulcers may or may not be surrounded by inflamed or red halos [51]. They usually resemble major aphthous ulcers and can influence any part of the oral mucosa, including the tongue and palate. These ulcers cause significant discomfort and typically heal with scarring in about 14 days [52]. Periodontal disease is commonly observed in individuals with cyclic neutropenia [54]. It can lead to severe destruction of supporting tissues and early loss of both primary and permanent teeth—even with regular medical and dental care [55].

The periodic decrease in neutrophil count—usually occurring approximately every 21 days and duration of 3–5 days—makes individuals susceptible to infections and results in oral symptoms. The best dental care for these individuals includes frequent monitoring and maintaining oral hygiene to prevent complications [56].

##### 2.1.5.2. Recurrent Herpes

Recurrent herpes simplex infection typically appears at the junction between the skin and mucosa of the lips [17]. In some patients, lesions may also occur inside the mouth, most often on the hard palate or attached gingiva [57].

Hours before the appearance of small red papules, an intense itching or burning sensation develops in the affected area. These papules transform into thin‐walled vesicles within a few hours. The vesicles often appear in small, unilateral clusters on keratinized mucosa, such as the hard palate or attached gingiva, and are accompanied by an underlying erythema. The lesions then dry out, crust over, and heal in 5–7 days without leaving a scar [57]. Clinical examination should be performed carefully as intraoral herpes infections can be difficult to distinguish from recurrent herpetiform aphthous ulcers or other viral ulcers. Factors like trauma, immune deficiencies (e.g., HIV infection or drug‐induced immunosuppression), menstruation, pregnancy, allergy, sun exposure, emotional stress, and upper respiratory tract infections may act as triggers for recurrent infections [17].

### 2.2. Exophytic Lesions

Exophytic lesions are defined as the result of pathologic overgrowth that passes through the normal contour of the oral mucosa. Hyperplasia, hypertrophy, neoplasia, and accumulation of body fluid can cause the over‐contoured shape of these lesions. Exophytic lesions were first categorized by surface texture: smooth or rough. Rough surfaces were further subclassified into seven different divisions (verrucous, papillomatous, and papillomatosis [pebbly or cobblestone]) [58]. The following section narrates six different sources of exophytic lesions on the gingiva or alveolar ridge (Figure 2).

#### 2.2.1. Congenital/Developmental

##### 2.2.1.1. Hereditary Gingival Fibromatosis (HGF)

HGF is a rare, slowly progressive condition characterized by genetically driven gingival overgrowth, most often inherited in an autosomal dominant manner. Notably, Cowden syndrome and Bardet–Biedl syndrome are strongly associated with gingival overgrowth. HGF may also coexist with aggressive periodontitis, underscoring the multifactorial interplay of genetic and environmental factors in gingival diseases [8, 59]. HGF is classified among nonplaque‐induced gingival diseases and typically presents clinically as a generalized fibrotic enlargement of the gingiva, particularly affecting the maxillary tuberosities, anterior gingiva, and retromolar pads [8]. When generalized fibrous overgrowth of the gingiva is observed, HGF should be included in the differential diagnosis, along with other entities such as drug‐induced gingival overgrowth and other forms of fibrous gingival hyperplasia [60, 61]. For definitive diagnosis, excisional biopsy and subsequent histopathological analysis are often indicated [8].

#### 2.2.2. Inflammatory Hyperplastic Lesions

##### 2.2.2.1. Specific Lesions

In this review, specific lesions refer to those that occur solely on the gingiva and alveolar ridge area.

###### 2.2.2.1.1. Peripheral Giant Cell Granuloma (PGCG)

PGCG is a reactive lesion typically originating from the marginal gingiva, connective tissue of the periosteum, or the periodontal ligament. PGCG is frequently observed in females, shows a predilection for the mandible, and commonly presents on the gingiva or alveolar ridge. According to one study, PGCG was identified as the most prevalent lesion among reactive oral lesions [8, 58, 62–65]. Clinically, PGCG may appear as a sessile or pedunculated lesion with a nodular or ulcerated surface. The lesion typically appears red to purple or even bluish‐brown, with a usual diameter of less than 2 cm. A characteristic “dumb‐bell” shape may be seen when the lesion extends through interproximal contact points [8, 58, 60, 62, 66]. In the differential diagnosis of fibrous gingival hyperplasia, PGCG should be distinguished from vascular epulis (pyogenic granuloma [PG]) and fibrous epulis. When multiple lesions are present, underlying systemic conditions such as hyperparathyroidism should also be considered [58, 60, 67–69]. Surgical excision and histopathological assessment remain the main treatment approach, serving as diagnostic and therapeutic approaches [58, 69, 70] (Figure 6).

**Figure 6:**
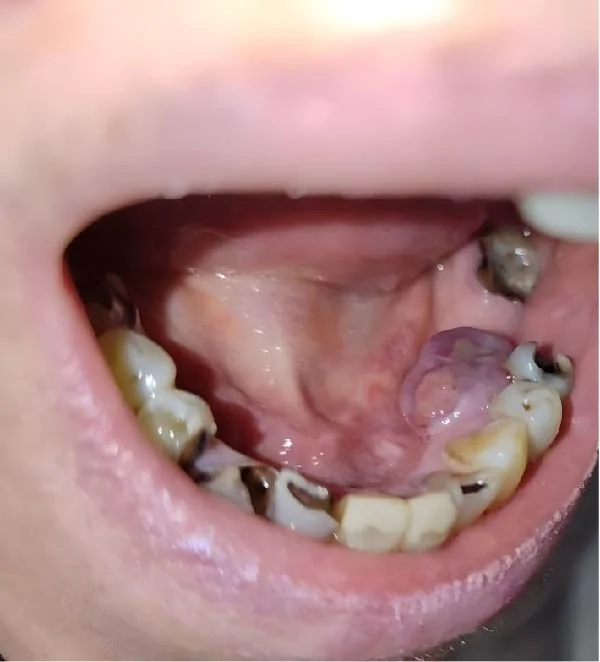
Peripheral giant cell granuloma with ulcerated surface in the posterior portion of the mandible.

###### 2.2.2.1.2. Peripheral Ossifying Fibroma (POF)

POF is a reactive, gingiva‐associated lesion that typically arises from the interdental papilla or the periodontal ligament, with a predilection for the anterior maxilla. It is most often observed in female patients, particularly in the second decade of life, with a diameter of usually less than 1.5 cm. In some cases, POF may develop as a sequela of a long‐standing PG [8, 58, 62, 71, 72]. Clinically, POF typically presents as a smooth, firm, red‐to‐pink mass that may be pedunculated or sessile. Though uncommon, tooth mobility and underlying bone resorption may occur in larger or more advanced cases [8, 58, 73]. Histopathologically, POF is characterized by a stratified squamous epithelial covering over a cellular fibrous connective tissue stroma, which often contains areas of calcification, and occasionally, multinucleated giant cells may be observed [62]. During differential diagnosis, POF should be distinguished from other gingival enlargements, particularly PG [67, 70]. The gold standard management is complete surgical excision followed by histological evaluation to confirm the diagnosis and rule out other pathologies [8, 58] (Figure 7).

**Figure 7:**
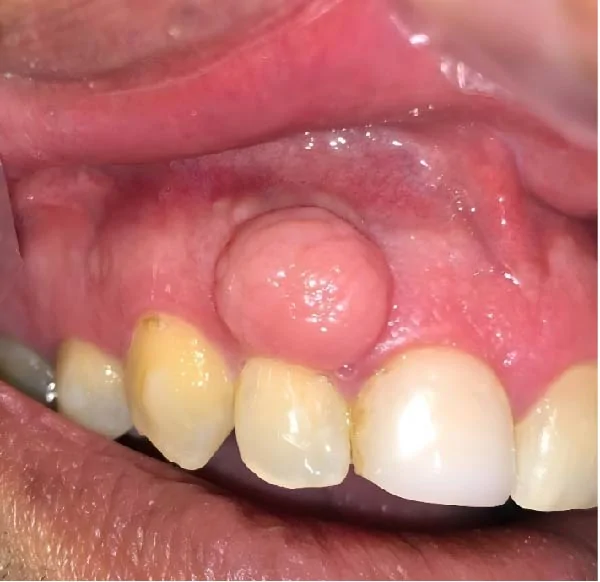
Peripheral ossifying fibroma with a smooth surface in the anterior portion of the maxilla.

##### 2.2.2.2. Nonspecific Lesions

In this article, nonspecific lesions refer to those that occur on multiple areas, including the gingiva and the alveolar ridge.

###### 2.2.2.2.1. Pyogenic Granuloma (PG)

PG is a reactive vascular lesion commonly found on the gingiva. It typically arises in response to local trauma, dental calculus, chronic irritation, or certain medications. Clinically, PG presents as a soft tissue mass involving the gingiva, pink to red or purplish in color. It may appear smooth or lobulated and be either pedunculated or sessile [8, 59, 61, 68, 69]. Histologically, PG demonstrates lobular capillary proliferation, characterized by endothelial cell proliferation. Surface ulceration and inflammatory infiltration may also be present [60, 62, 67, 68, 74]. Excisional biopsy followed by histopathological analysis remains the standard treatment. Additionally, minimally invasive methods (e.g., laser therapy and cryotherapy) have been proposed as adjuncts. However, recurrence is not uncommon, especially if the irritating factor is not eliminated [8, 58, 67, 68] (Figure 8).

**Figure 8:**
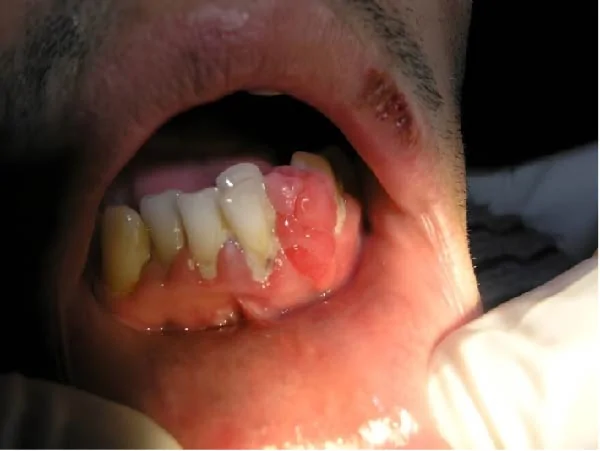
Pyogenic granuloma as an exophytic lobulated lesion on the anterior portion of the mandibular gingiva.

###### 2.2.2.2.2. Irritation Fibroma

Inflammatory fibrous hyperplasia (IFH), also known as irritation fibroma, represents a common benign reactive lesion of the oral mucosa, accounting for up to 40% of mucosal pathologies. It demonstrates a female predilection and frequent manifestation in patients in the sixth decade of life. The occlusal line of the buccal mucosa is the most commonly affected site [8, 58, 60, 62]. Clinically, IFH appears as exophytic, smooth, pink, nodular masses of a fibrous consistency. While most often arising from the interdental papilla, lesions can also be found in other intraoral locations. They may be sessile or pedunculated, typically exhibit a color similar to the adjacent mucosa, and in some instances, display a dumbbell‐shaped morphology, similar to that seen in PGCG [8, 58, 60, 62, 71, 72]. Differential diagnosis includes PG, PGCG, hereditary gingival, lesions associated with SOS1 gene mutations [59, 60, 67, 68]. Histopathological evaluation reveals hyperplastic keratinized stratified squamous epithelium overlaying a nodular mass of fibrous connective tissue [60, 62]. The recommended treatment includes conservative excisional biopsy, followed by histological analysis to confirm diagnosis and rule out other lesions [8, 58, 62].

###### 2.2.2.2.3. Giant Cell Fibroma (GCF)

GCF is a rare inflammatory fibrous hyperplastic lesion, accounting for less than 5% of fibrous lesions. Showing a slight female predilection, it is commonly found in the mandible. However, its exact etiology remains unclear [58, 75, 76]. Clinically, GCF appears as a firm, pink, tumor‐like mass that may be either pedunculated or sessile, with a rough surface exhibiting papillary or granular characteristics [17, 77]. Histological assessment reveals large fibroblasts and stellate in shape [78]. Excisional biopsy with a surgical blade, electrosurgery, or a laser is the recommended approach, with histopathological examination essential for a definitive diagnosis [58].

###### 2.2.2.2.4. Pregnancy Tumor

This lesion is associated with hormonal changes during pregnancy and typically emerges in the first trimester. Some researchers suggest that individuals with good oral hygiene are unlikely to develop this condition [58, 60, 67]. Clinically, it closely resembles PG as previously described, though in some instances, it may mimic an irritation fibroma [58]. The differential diagnosis includes PG, PGCG, POF, IFH, leukemia, granulomatosis with polyangiitis, certain early‐stage lymphomas, and rare metastatic tumors such as sarcomas, which can clinically resemble PG [8, 60, 67, 68, 79–82]. The approach to surgical treatment remains controversial. While some authors advocate excision during pregnancy, in the case of asymptomatic and nonbleeding ones, it is recommended to monitor the lesion until after birth delivery [58, 67].

#### 2.2.3. Neoplastic

##### 2.2.3.1. Acute Myelocytic Leukemia

The most common type of leukemia involving the gingiva is acute myeloid leukemia (AML), particularly of monocytic origin [8, 58, 83]. When localized, it may be clinically mistaken for a PG or a giant cell epulis [60]. The preferred diagnostic approach involves a differential blood cell count from venous blood, along with a bone marrow biopsy [8] (Figure 9).

**Figure 9:**
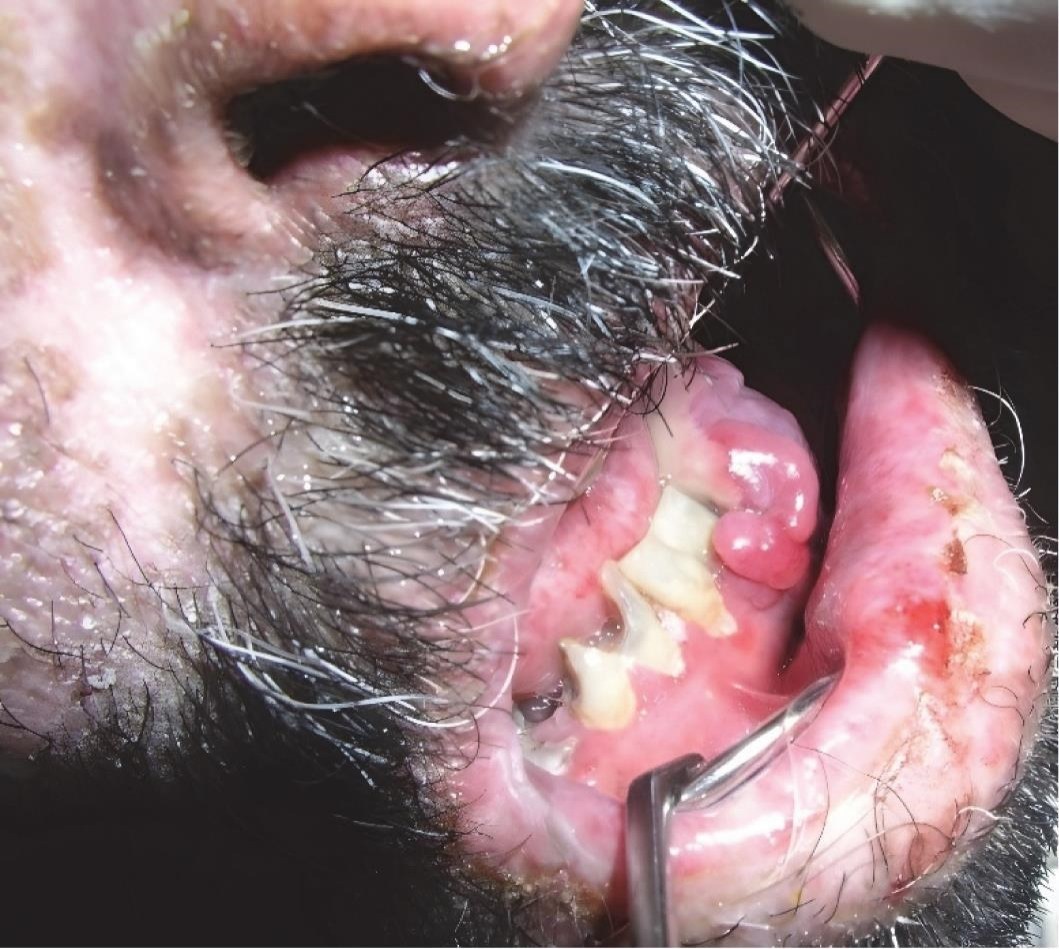
Gingival enlargement in a patient with acute myelocytic leukemia.

##### 2.2.3.2. SCC

SCC rarely appears on the gingiva, causing misinterpretations as being tooth‐related abscess or inflammatory conditions that can delay the accurate diagnosis [33]. One of the possible representations of SCC is the exophytic form. This usually shows a rough surface, with prominent signs including a large mass with irregular margins and possible ulcerations on the surface caused by local irritations [58]. More details regarding its management and recurrence have been mentioned in the previous section under the same category name (Figure 10).

**Figure 10:**
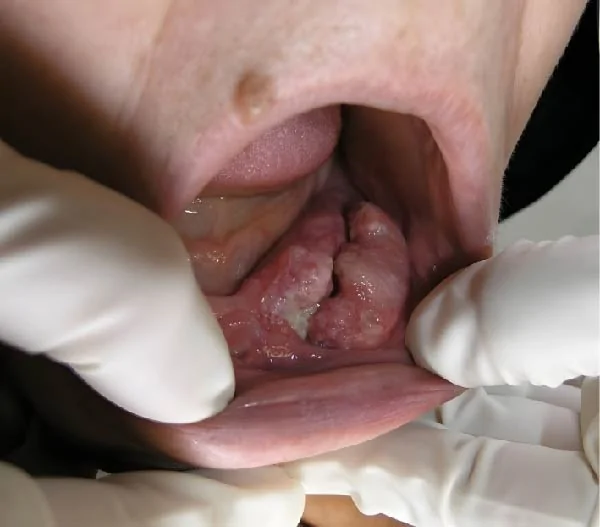
Squamous cell carcinoma with ulcerated surface on mandibular alveolar ridge.

#### 2.2.4. Metastatic Lesions

Metastasis represents the terminal phase of cancer progression, characterized by the dissemination of malignant cells to distant organs or tissues [84]. In patients with a prior diagnosis of malignancy, the possibility of oral metastasis should always be considered during the clinical evaluation [60].

Although the oral cavity is an uncommon site for metastatic spread, the gingiva is the most frequently affected area among the oral soft tissues. In most cases, the metastases do not involve the underlying alveolar bone. Metastatic tumors in the oral region are typically diagnosed in patients during their 6^th^ and 7^th^ decades of life, with a higher incidence in males. The jaws appear to be equally affected, and no significant difference has been observed between the anterior and posterior regions of the jaws.

The lungs represent the most common primary site, followed by the kidneys, skin, and breasts [84]. Additionally, metastases originating from the gastrointestinal tract and prostate have also been reported [60]. The distribution of primary tumor sites varies by gender, and the majority of originating malignancies are of epithelial origin. Common symptoms include swelling, pain, and spontaneous bleeding. According to Allon et al. [84], the clinical presentation was predominantly exophytic in nature (96%), with ~24% of them showing ulceration.

Differential diagnosis should consider benign peripheral tumor‐like lesions such as PG, PGCG, PGCG, epulis, and hemangioma. An important clinical correlation has been reported between dentition and metastasis location. Among dentate patients, 80% of metastatic lesions were found in the gingiva. In contrast, edentulous individuals exhibited a lower gingival involvement rate, with lesions more commonly located on other mucosal surfaces, particularly the tongue [84].

#### 2.2.5. Granulomatous Disease

##### 2.2.5.1. Wegener’s Granulomatosis

Granulomatosis with polyangiitis, previously known as Wegener’s granulomatosis, is a rare systemic vasculitis closely linked to antineutrophil cytoplasmic antibodies (ANCA). Although its exact etiology remains unclear, it is believed to result from an abnormal immune response to environmental or infectious triggers in genetically predisposed individuals. The disease is typically diagnosed between the ages of 45 and 60, with a higher prevalence in white populations [68].

Oral manifestations can occasionally serve as the initial indication of this condition and may remain localized for extended periods before systemic involvement develops [85]. While the respiratory tract is the most commonly affected site, this entity can involve nearly any organ [60].

Oral lesions are observed in ~2% of patients [84]. The hallmark oral manifestation is “strawberry gingivitis,” appearing as red, nodular lesions with an irregular surface and a tendency to bleed [60]. These changes often begin in the interdental papillae and can gradually extend across the entire attached gingiva. Other oral signs may include persistent oral ulcers, alveolar bone destruction, tooth mobility, and the formation of oroantral fistulas [86].

Although the clinical appearance of its gingival lesions is distinctive, differential diagnosis should include other vascular or granulomatous conditions, especially when lesions are localized. Definitive diagnosis from small gingival biopsies can be challenging. Therefore, other granulomatous diseases such as tuberculosis, deep fungal infections, Crohn’s disease, and sarcoidosis must be excluded. For a definitive diagnosis, serologic testing for PR3‐ANCA is advised [60].

##### 2.2.5.2. Sarcoidosis

Sarcoidosis is a systemic granulomatous condition of unknown origin, characterized by noncaseating granulomas affecting multiple organs. The peak incidence occurs in adults aged 20–40 years, with a slight female predominance. Although the exact cause remains unknown, potential contributing factors include infectious agents, environmental exposures, and autoimmune mechanisms.

Around 90% of patients show abnormal chest radiographic findings at some point during the illness, which count the lungs as one of the most common involved sites [87]. Oral manifestations are rare. When present, gingival involvement may appear as an exophytic lesion [88]. Salivary gland involvement is relatively common, frequently reported in the form of xerostomia [89]. The diagnosis of sarcoidosis is typically made based on a combination of clinical, radiographic, and histopathological findings [87].

#### 2.2.6. Drug Induced Gingival Overgrowth

This phenomenon is a pathological gingival enlargement resulting from excessive connective tissue growth. It predominantly affects the anterior regions of both jaws. Although more than 20 medications have been implicated, it is most commonly linked to calcium channel blockers (CCBs), immunosuppressive drugs, and antiepileptic medications [90]. Risk factors for this entity include genetic predisposition, male gender, poor oral hygiene, and existing gingival inflammation [91]. During the initial stages, the condition is characterized by noninflamed pink, lobulated, and thickened gingival tissues. As the disease progresses, gingiva may become inflamed and develop as a reddish or bluish discoloration with bleeding episodes [90].

The primary goal of treatment is achieving acceptable esthetic outcomes and alleviating pain and discomfort. Management strategies may be either conservative or surgical in nature [90]. Nonsurgical options are generally the first line of treatment that include meticulous oral hygiene practices, mechanical plaque removal, and, most importantly, discontinuation of the causative medication [92]. In cases involving CCBs, discontinuing the drug or switching to an alternative antihypertensive one often leads to significant improvement. However, this substitution may not always be possible, particularly in patients with poorly controlled hypertension [93].

If conservative measures fail, surgical intervention becomes necessary. This may involve gingivectomy or periodontal flap surgery. The use of carbon dioxide (CO_2_) lasers has emerged as a newly emerged technique, offering effective tissue removal along with excellent postoperative hemostasis. Nevertheless, studies indicate that up to 40% of patients who continue the causative medication postsurgery may experience recurrence [90].

### 2.3. White Lesions

White lesions represent a wide variety of lesions with different etiologies. The first issue to consider is whether the lesion can be scraped off or not. Based on this finding, they are initially classified as scrubbable and unscrubbable lesions. In the case of unscrubbable ones, lesions with keratin layer thickening with multiple patterns (papular, reticular, and annular) or even nonpattern ones should be assessed [1] (Figure 3a).

#### 2.3.1. Scrubbable

##### 2.3.1.1. Pseudomembranous Candidiasis

Oral candidiasis is the most prevalent fungal infection of the oral cavity caused by *Candida albicans*. The acute form of oral candidiasis is often seen in infants due to their immature immune systems, in the elderly due to weakened immune systems, and in patients receiving broad‐spectrum antibiotics [54]. Clinical manifestations of the lesion are creamy white plaques, spots, and papules that can be easily removed, leaving an erythematous and hemorrhagic surface. The chronic form of oral candidiasis is clinically indistinguishable from the acute one and is more common in patients with HIV infection and patients using inhaled steroid sprays. The buccal mucosa is the commonly affected site in the oral cavity, followed by the tongue and palate [41].

##### 2.3.1.2. Debris/Materia Alba

Materia alba refers to a microbial aggregation, desquamated epithelial cells, leukocytes, and food remnants. This structure does not display the organized internal structure observed in the microbial plaque. Materia alba is a soft, sticky, yellowish, or grayish‐white accumulation that is slightly less adhesive than dental plaque. Gingival tissue irritation potential is mostly due to the presence of bacteria and their metabolic products [94].

#### 2.3.2. Unscrubbable

##### 2.3.2.1. Solitary

###### 2.3.2.1.1. Frictional Keratosis

Frictional or traumatic keratosis is conceptualized as white plaques with a rough, threadlike surface that are articulately associated with a recognizable cause of mechanical irritation. These lesions have the potential to display in dysplastic leukoplakia forms. So far, no evidence of malignancy change in traumatic keratosis has been reported. If the irritation is eliminated, the lesion resolves completely within 2 weeks [95]. Mild mechanical trauma induced by toothbrushing may lead to gingival hyperkeratosis, appearing as a white lesion with a leukoplakia‐like lesion [96].

###### 2.3.2.1.2. Oral Leukoplakia

Oral leukoplakia is characterized as a white spot or plaque. The reported global prevalence of this lesion is 4.11%, with the highest occurrence in Asia [96, 97]. Most lesions are prevalent among people in their 6^th^ decade of life. Oral leukoplakia is known as the most prevalent potentially malignant lesion in the oral cavity, with this being the underlying cause in ~16%–62% of cases of SCC. Although no etiology has been confirmed for this lesion, some researchers have suggested a relationship between this lesion and several factors, including tobacco use, alcohol, Sanguinaria, ultraviolet radiation, mechanical trauma, genetic factors, and microorganisms [98, 99]. Clinically, oral leukoplakia appears as an irreversible white, nonremovable, slightly elevated plaque that may have a wrinkled, leathery, and sometimes dry, cracked‐mud appearance. Based on color and appearance, leukoplakia is divided into 2 main subgroups: homogenous and nonhomogenous. Homogenous leukoplakia presents uniform patches with thin, flat, or wrinkled surfaces, while nonhomogenous ones show a mixed appearance of nodular and verrucous textures [97]. The most prevalent location for these lesions are the buccal mucosa, lip vermilion, and gums [40].

###### 2.3.2.1.3. SCC

SCC is a disease with a multifactorial etiology. Well‐known factors include tobacco use, alcohol, contact with phenol, viral, bacterial, and fungal infections, electrogalvanic reactions, radiation, genetic factors, immunodeficiency, malnutrition and iron deficiency anemia, and hereditary diseases. Clinical manifestations consist of red/white color with granular, rough, warty, fungal, papillary, or scaly surface texture that display as irregular masses or ulcers with a rolled margin and firmness on palpation. High‐risk areas in oral cavity are mainly the floor of mouth, posterior lateral border, and inferior surface of the tongue. The 5‐year survival rate for SCC has been reported to be 55% [96, 100].

###### 2.3.2.1.4. Verrucous Carcinoma (VC)

VC is a rare variant of oral SCC that has well‐defined clinical and pathological features and accounts for ~2%–9% of all oral carcinomas [101]. It is commonly observed in older men over the age of 55. The progression rate of the lesion is slow. Despite local invasion, VC has a low tendency to metastasize [102]. Several precancerous lesions, including leukoplakia, erythroplakia, and proliferative varicose leukoplakia, can develop into varicose carcinoma over time. Tobacco use, alcohol consumption, and smokeless tobacco have been reported as possible causative factors for this lesion. VC appears as a thick, white plaque with well‐defined borders and has a papillary or wart‐like surface. Although all areas in the oral cavity can be affected by this lesion, the vestibular area of the mandible, gums, tongue, and hard palate are the more commonly reported sites. The malignant change rate of VC has been reported to be 1%–16%, which is mostly observed in the tongue [103].

##### 2.3.2.2. Multiple

###### 2.3.2.2.1. Proliferative Verrucous Leukoplakia (PVL)

The World Health Organization defines PVL as a rare but specific form of precancerous lesions in the oral cavity [104]. The average age of patients with persistent PVL lesions has been reported to be over 60 years old.

Although the definitive etiology of this lesion is unknown, genetic factors and viral infections, especially human papillomavirus (HPV 16 and HPV 18) and Epstein‐Barr virus (EBV), have been suggested as possible causes [105]. In the early stages, PVL manifests as small, well‐defined white plaques or spots that appear as localized, homogeneous keratotic lesions. These lesions gradually enlarge and involve a broad surface of the oral mucosa. Simultaneously, multifocal, heterogeneous areas with a rough, mottled surface may appear, which present as exophytic, warty, verrucous, polypoid, or erythematous forms of involvement [106]. PVL usually appears bilaterally, with the most common sites being the buccal mucosa, gums, and alveolar regions. The gums are the most commonly affected, and a subtype of this disease has been introduced as proliferative verrucous gingival leukoplakia [95]. The rate of malignancy in PVL has been reported to range from 63.3% to 100% [104] (Figure 11).

**Figure 11:**
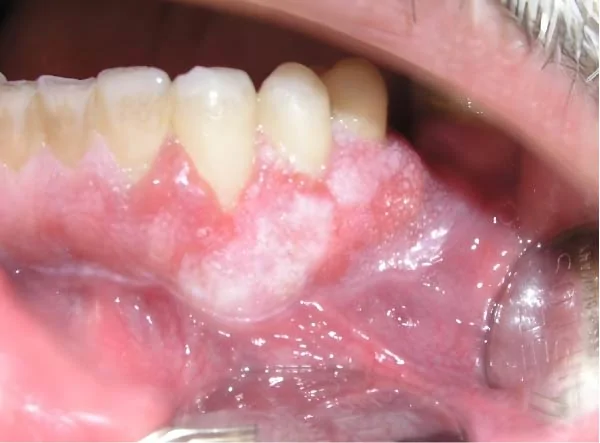
Unscrubbable white lesion on mandibular ridge in patient with use of smokeless tobacco.

###### 2.3.2.2.2. VC

This lesion can also be represented in multiple forms. More details have been discussed in the previous section with the same name.

###### 2.3.2.2.3. Frictional Keratosis

Another representation of this lesion is in its multiple forms. The lesion is thoroughly discussed in the previous section.

### 2.4. Pigmented Lesions

Pigmented lesions are brown‐to‐black discolorations that result either from exogenous depositions or endogenous pigments. Pigmentated lesions range from a sole to multiple lesions, with a pinpoint to diffuse pattern. The last section discuss about lesions in this category with possible etiologies [107] (Figure 3b).

#### 2.4.1. Physiologic Pigmentation

Physiological pigmentation of the oral mucosa, also known as racial pigmentation, is a common and anatomic variation particularly prevalent among individuals with darker skin tones [108]. The known etiologic factor is increased melanocyte activity rather than an increased number of melanocytes [109] (Figure 12).

**Figure 12:**
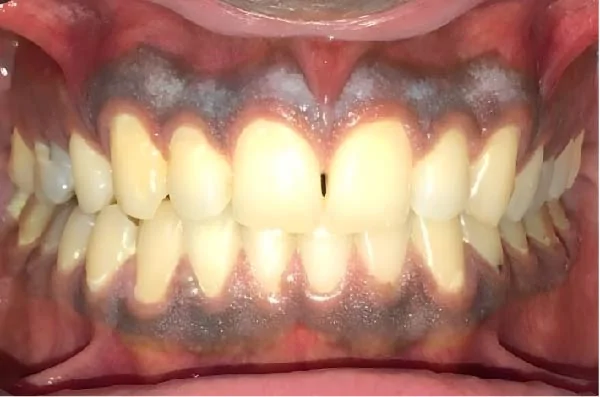
Physiologic pigmentation in both upper and lower gingiva in a patient with no history of smoking, medication use, or systemic disease/condition.

This pigmentation can be observed at any age with no gender predilection, although its prevalence increases with age [110]. This increase may be due to the cumulative effects of stimuli such as minor functional trauma, inflammation, medications, or tobacco smoke [108]. Physiologic oral pigmentation appears as asymptomatic brown macules, either solitary or multiple, with well‐defined borders. These spots can occur on any part of the oral mucosa, but the gingiva is the most commonly affected site [107, 108].

Gingival pigmentation usually appears in a bilateral and symmetrical pattern and typically does not extend beyond the mucogingival junction. This type of pigmentation generally appears gradually during the first two decades of life, and many affected individuals may not be aware of its presence [108].

#### 2.4.2. Amalgam Tattoo

An amalgam tattoo is a relatively frequent occurrence in the oral mucosa, appearing as an asymptomatic bluish grey macule or plaque with indistinct margins. It commonly develops along the gingival margin and the alveolar mucosa. Amalgam particles may inadvertently or traumatically become embedded in the submucosal tissue during dental procedures such as crown placement, removal of old amalgam restorations, tooth extractions of previously restored teeth, endodontic therapy, or even flossing shortly after restorative treatment [111, 112] (Figure 13).

**Figure 13:**
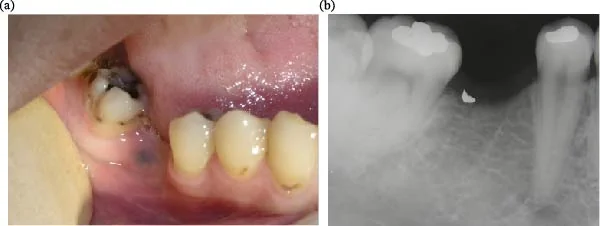
(a) Clinical manifestation of an amalgam tattoo as a pigmented lesion in the posterior portion of the mandibular ridge. (b) Radiographic appearance of the remaining amalgam particle in the alveolar soft tissue.

In some cases, metal ions from amalgam can be released due to galvanic interactions between dissimilar alloys in the oral cavity (e.g., a gold restoration adjacent to an amalgam‐filled tooth). Mercury from the amalgam may diffuse through soft tissues, ultimately resulting in the formation of an amalgam tattoo [113]. Gingiva and alveolar mucosa are the most frequently involved areas, with the mandible being more commonly affected than the maxilla [112, 113].

Different tissue responses to amalgam might be observed in the oral cavity. They may include macrophage activity or chronic inflammation, often manifesting as a foreign body reaction or, in some cases, no tissue reaction at all. Generally, no treatment is required for amalgam tattoos. However, when radiographic evidence of radiopaque particles is absent, especially in ambiguous pigmented lesions, a biopsy is recommended to exclude more serious conditions [112].

#### 2.4.3. Malignant Melanoma

Oral melanoma manifests as a primary neoplasm or presents as a metastasis, most commonly from a cutaneous origin. Clinical presentation of melanoma may vary from a solitary, benign‐appearing pigmented macule to more concerning lesions exhibiting irregular margins or exophytic growth patterns [81].

During the initial stages, mucosal melanoma may mimic other pigmented lesions, such as a melanotic macule, melanocytic nevus, or even an amalgam tattoo [112]. In some cases, multiple dispersed pigmented lesions may be observed. Rarely, nonpigmented (amelanotic) variants can occur. The peak incidence is reported in individuals in their sixth decade of life [114]. The most commonly affected intraoral sites are the hard palate and maxillary gingiva, which together account for ~2/3 of cases [81, 112].

Because of the aggressive behavior and the tendency for late detection, oral melanoma is associated with a poor prognosis [115]. The primary treatment modality is surgical removal [116]. Recurrence rates are high (85%), and distant metastases may develop in half of the patients. Hence, lifelong monitoring is essential for patients diagnosed with oral melanoma [115].

#### 2.4.4. Melanotic Macule

Melanotic macules are the most common oral mucosal lesions originating from melanocytes [117]. These small, solitary lesions with well‐defined borders and often uniform pigmentation typically occur in adult women around the age of 43. Although any area of the oral mucosa may be affected, the lower lip, gingiva, and palate are the most commonly involved sites [117, 118].

Trauma or local injury can be a possible cause, although this has not yet been definitively established. Melanotic macules rarely exceed 1 cm in diameter. Although these lesions are benign in nature, a biopsy is usually required for a definitive diagnosis because mucosal melanoma can appear clinically similar to melanotic macules [118].

Once a diagnosis is confirmed, no further treatment is necessary. The lesion neither recurs nor exhibits malignant changes. Since most melanotic macules appear on the lip, patients often request complete removal due to esthetic demands [118]. These macules result from the increased functional activity of regional melanocytes, leading to elevated levels of melanin production. Histologically, this lesion is characterized by abundant melanin pigment in the basal cell layer of the epithelium and the presence of melanin leakage in the superficial submucosal areas [81, 119].

## 3. Discussion and Conclusion

Recent reports have indicated that oral cavity cancers occur in 10.2 people per 100.000. Many of these lesions can appear in a chronic, progressive form that negatively impacts patients’ quality of life and, at the same time, highlights the importance of early intervention and management [117]. On the other hand, various cancers can be initially diagnosed by examining abnormalities in the oral mucosa. Although not all oral lesions are malignant, early diagnosis and management may improve the prognosis [97]. The gingiva and underlying alveolar ridge are important parts of the oral cavity. Lesions in these regions include a variety of entities with different clinical manifestations and specific therapeutic approaches. In the present review, we propose a practical decision tree for gingival and alveolar ridge lesions, as well as a concise review of each entity.

The first group comprises oral ulcers and erosions. Oral ulcers are typically classified into 3 main groups: 1/ Acute lesions with sudden commencement and short duration, 2/ Chronic ulcers with slow onset and progression, and 3/ Recurrent lesions [120]. All these subgroups can be presented in single or multiple forms. While the main etiologic factors for acute lesions are viruses, bacteria, and allergens, multiple chronic lesions are mainly caused by autoimmune factors or carcinoma [7].

The second category exhibited exophytic lesions. Congenital disorders, reactive lesions, drug‐induced hyperplasia, acute neoplasms, metastatic lesions, and granulomatosis are among the most prevalent possible etiologies. Since therapeutic interventions vary considerably, precise physical examination (color, size, texture, location, duration of presence, rate of progression) and complete history assessment should be performed to select the best treatment approach [57].

The third category comprises white lesions, initially divided into scrubbable and unscrubbable. A clinical view of a white lesion can result from either a thickened keratotic layer or the accumulation of exogenous material. One of the most important parts in diagnosing a white lesion is the possibility of scraping the white layer off the underlying tissue ^1^. Other pathologic findings, including surface appearance, time of presentation, and local risk factors, should be carefully examined for a precise diagnosis [121].

The last subgroup of the decision tree belongs to pigmented lesions. Pigmented lesions represent a vast range of clinicopathologic features, from nonmalignant conditions like physiologic pigmentation and amalgam discoloration to more serious ones like melanoma. Many pigmented lesions can be diagnosed based on their shape, size, presence of an irritating factor, and history. A practical maneuver should be planned for each patient based on the final diagnosis [122].

The oral cavity is an under‐examined area during general practice. Multiple etiologies, including reactive, inflammatory, and neoplastic ones, can be the main cause of oral lesion manifestations on different parts of the oral mucosa [123]. Though malignant lesions usually show a specific topographic predilection, the entire oral mucosa can be exposed to all types of trauma. This difference between the reactive and malignant lesions highlights the importance of precise description of the biopsy site and the lesions’ appearance [124].

However, the proposed decision tree should be applied in clinical practice with caution. This diagnostic algorithm primarily focuses on the clinical signs and manifestations of intraoral lesions. Because diverse lesions with distinct definitive diagnosis may exhibit similar clinical presentations, incorporating histological features is crucial. Furthermore, to avoid misinterpretation, careful attention should be paid to patients’ medical history, the progression pattern of the lesion, and imaging or biopsy findings, when indicated.

The main objective of the present decision tree is to simplify the process of a differential diagnosis list preparation. During the initial examination, clinicians frequently encounter conditions with overlapping clinical manifestations. This decision tree aims to mitigate diagnostic ambiguity by guiding clinicians in establishing a preliminary list of possible lesions based on clinical features, which can be subsequently refined using histologic results.

This simplification can also help in the management of patients with oral potentially malignant disorders, which mainly require a multidisciplinary approach [125]. To validate the diagnostic accuracy of the current proposed algorithm, future research articles, including prospective multicenter clinical validation studies assessing diagnostic accuracy, interobserver agreement, and real‐world applicability, are highly recommended to evaluate its efficacy in clinical practice.

In conclusion, in this review, we categorized gingival and alveolar ridge lesions into four main subgroups of ulceration/erosion, exophytic, white, and pigmented lesions to facilitate diagnosis with simultaneous high accuracy. Differential diagnosis of these lesions requires a stepwise approach; so, an updated decision tree was also proposed to help clinicians to come to a reasonable diagnosis without loss of time, as well as appropriate treatment modality selection.

## Author Contributions

Conceptualization: Hamed Mortazavi. Data curation: Shaghayegh Khoshgou, Amirsalar Mofid, and Ehsan Chegeni. Formal analysis: Aida Kheiri, Shaghayegh Khoshgou, and Amirsalar Mofid. Investigation: Hamidreza Eghbalikhosro, Zahra Karami, and Ehsan Chegeni. Methodology, supervision, visualization: Hamed Mortazavi and Aida Kheiri. Project administration: Shaghayegh Khoshgou, Amirsalar Mofid, Ehsan Chegeni, and Aida Kheiri. Resources: Amirsalar Mofid and Shaghayegh Khoshgou. Validation: Hamed Mortazavi, Aida Kheiri, Shaghayegh Khoshgou, and Amirsalar Mofid. Writing – original draft: Aida Kheiri, Hamidreza Eghbalikhosro, Zahra Karami, and Ehsan Chegeni. Writing – review and editing: Amirsalar Mofid, Zahra Karami, Hamidreza Eghbalikhosro, and Ehsan Chegeni.

## Funding

This study did not receive any specific funding.

## Disclosure

All authors have read and approved the final version of the manuscript. Aida Kheiri had full access to all of the data in this study and takes complete responsibility for the integrity of the data and the accuracy of the data analysis.

## Ethics Statement

This is a review article, and ethical approval was not required.

## Consent

The authors have nothing to report.

## Conflicts of Interest

The authors declare no conflicts of interest.

## Data Availability

The authors confirm that all data are presented in the manuscript file.

## References

[1] Mortazavi H. , Safi Y. , Baharvand M. , Jafari S. , Anbari F. , and Rahmani S. , Oral White Lesions: An Updated Clinical Diagnostic Decision Tree, Dentistry Journal. (2019) 7, no. 1, 10.3390/dj7010015, 15. PMC6473409 30736423

[2] Maymone M. B. , Greer R. O. , and Burdine L. K. , et al.Benign Oral Mucosal Lesions: Clinical and Pathological Findings, Journal of the American Academy of Dermatology. (2019) 81, no. 1, 43–56, 10.1016/j.jaad.2018.09.061. 30447312

[3] Pachpande P. S. , Mandale M. S. , Bhavthankar J. D. , Humbe J. G. , and Zanwar P. R. , Red and White Lessions of Oral Cavity a Clinical Perspective for Diagnosis and Treatment, Indian Journal of Pathology and Oncology. (2023) 10, no. 3, 233–238, 10.18231/j.ijpo.2023.052.

[4] Vilchis M. D. , López-Ríos P. , García I. M. , and Cepeda L. A. , Impact of Oral Mucosa Lesions on the Quality of Life Related to Oral Health. An Etiopathogenic Study, Medicina Oral Patologia Oral y Cirugia Bucal. (2016) 21, 8. 10.4317/medoral.20866 PMC4788796 26827060

[5] Alblowi J. A. and Binmadi N. O. , Histopathologic Analysis of Gingival Lesions: A 20-Year Retrospective Study at One Academic Dental Center, Journal of Clinical and Experimental Dentistry. (2018) 10, no. 6, 10.4317/jced.54766, e561. PMC6005087 29930774

[6] Rashid H. , Lamberts A. , and Diercks G. F. , et al.Oral Lesions in Autoimmune Bullous Diseases: An Overview of Clinical Characteristics and Diagnostic Algorithm, American Journal of Clinical Dermatology. (2019) 20, no. 6, 847–861, 10.1007/s40257-019-00461-7. PMC6872602 31313078

[7] Mortazavi H. , Safi Y. , Baharvand M. , and Rahmani S. , Diagnostic Features of Common Oral Ulcerative Lesions: An Updated Decision Tree, International Journal of Dentistry. (2016) 2016, 10.1155/2016/7278925, 7278925. PMC5066016 27781066

[8] Holmstrup P. , Plemons J. , and Meyle J. , Non–plaque-Induced Gingival Diseases, Journal of Clinical Periodontology. (2018) 45, no. S20, S28–43, 10.1111/jcpe.12938. 29926497

[9] Ahmadi-Motamayel F. , Najar-Karimi F. , Safabakhsh D. , Abbasi F. , Atapour P. , and Shahabi L. , A Simple Approach for the Diagnosis of Oral Ulcers: A Novel Algorithm, Avicenna Journal of Dental Research. (2024) 16, no. 2, 97–107, 10.34172/ajdr.1741.

[10] Fitzpatrick S. G. , Cohen D. M. , and Clark A. N. , Ulcerated Lesions of the Oral Mucosa: Clinical and Histologic Review, Head and Neck Pathology. (2019) 13, no. 1, 91–102, 10.1007/s12105-018-0981-8. PMC6405793 30701449

[11] Axéll T. and Koch G. , Traumatic Ulcerative Gingival Lesion, Journal of Clinical Periodontology. (1982) 9, no. 3, 178–183, 10.1111/j.1600-051X.1982.tb02057.x. 6954160

[12] Compilato D. , Corsello G. , and Campisi G. , An Unusual Traumatic Ulceration of the Tongue, Journal of Paediatrics and Child Health. (2012) 48, no. 12, 1104–1105, 10.1111/jpc.12002. 23217045

[13] Atarbashi-Moghadam F. , Atarbashi-Moghadam S. , Marashi S. A. , and Amid R. , Plasma Cell Gingivitis: A Systematic Review and Guideline to Diagnosis, Journal of Stomatology Oral and Maxillofacial Surgery. (2025) 126, no. 5, 10.1016/j.jormas.2025.102378, 102378. 40252807

[14] Shahrabi-Farahani S. and Aguirre S. , Herpesvirus-Related Lesions of the Oral Mucosa, Oral and Maxillofacial Surgery Clinics of North America. (2023) 35, no. 2, 175–187, 10.1016/j.coms.2022.10.012. 37019504

[15] Guttiganur N. , Devanoorkar A. , Aspalli S. , and Shetty S. , Herpes Zoster of Trigeminal Nerve After Dental Extraction, Indian Journal of Dental Research. (2013) 24, no. 3, 10.4103/0970-9290.118011, 396. 24025898

[16] Patil S. , Srinivas K. , Reddy B. S. , and Gupta M. , Prodromal Herpes Zoster Mimicking Odontalgia–A Diagnostic Challenge, Ethiopian Journal of Health Sciences. (2013) 23, no. 1, 73–77. PMC3613819 23559842

[17] Dommisch H. and Schmidt-Westhausen A. M. , The Role of Viruses in Oral Mucosal Lesions, Periodontology 2000. (2024) 96, no. 1, 189–202, 10.1111/prd.12553. PMC11579825 38411337

[18] Glick M. , Greenberg M. S. , Lockhart P. B. , and Challacombe S. J. , Ulceraive, Vesicular, and Bullous Lesions, Burket’s Oral Medicin, 2021, Wiley, 35–84.

[19] McCullough M. J. and Savage N. W. , Oral Viral Infections and the Therapeutic Use of Antiviral Agents in Dentistry, Australian Dental Journal. (2005) 50, no. s2, S31–5, 10.1111/j.1834-7819.2005.tb00382.x. 16416715

[20] Kawai K. , Gebremeskel B. G. , and Acosta C. J. , Systematic Review of Incidence and Complications of Herpes Zoster: Towards a Global Perspective, BMJ Open. (2014) 4, no. 6, 10.1136/bmjopen-2014-004833, e004833. PMC4067812 24916088

[21] Bhagat S. , Jaiswal P. , and Kotecha S. V. , Clinical Features, Etiopathogenesis, and Therapeutic Approaches of Acute Gingival Lesions: A Narrative Review, Cureus. (2024) 16, 10.7759/cureus.67129. PMC11407791 39290940

[22] Riley C. , London J. P. , and Burmeister J. A. , Periodontal Health in 200 HIV-Positive Patients, Journal of Oral Pathology & Medicine. (1992) 21, no. 3, 124–127, 10.1111/j.1600-0714.1992.tb00995.x. 1583595

[23] MacCarthy D. and Claffey N. , Acute Necrotizing Ulcerative Gingivitis Is Associated With Attachment Loss, Journal of Clinical Periodontology. (1991) 18, no. 10, 776–779, 10.1111/j.1600-051x.1991.tb00071.x. 1753002

[24] Williams C. A. , Winkler J. R. , Grassi M. , and Murray P. A. , HIV-Associated Periodontitis Complicated by Necrotizing Stomatitis, Oral Surgery, Oral Medicine, Oral Pathology, Oral Radiology. (1990) 69, 351–355. 10.1016/0030-4220(90)90298-7 2314860

[25] Coppola N. , Cantile T. , and Adamo D. , et al.Supportive Care and Antiviral Treatments in Primary Herpetic Gingivostomatitis: A Systematic Review, Clinical Oral Investigations. (2023) 27, no. 11, 6333–6344, 10.1007/s00784-023-05250-5. PMC10630243 37733027

[26] Piperi E. , Papadopoulou E. , and Georgaki M. , et al.Management of Oral Herpes Simplex Virus Infections: The Problem of Resistance. A Narrative Review, Oral Diseases. (2024) 30, no. 3, 877–894, 10.1111/odi.14635. 37279074

[27] Mancini A. , Inchingolo A. M. , and Marinelli G. , et al.Topical and Systemic Therapeutic Approaches in the Treatment of Oral Herpes Simplex Virus Infection: A Systematic Review, International Journal of Molecular Sciences. (2025) 26, no. 17, 10.3390/ijms26178490, 8490. PMC12428954 40943411

[28] Badwelan M. , Muaddi H. , Ahmed A. , Lee K. T. , and Tran S. D. , Oral Squamous Cell Carcinoma and Concomitant Primary Tumors, What Do We Know? A Review of the Literature, Current Oncology. (2023) 30, no. 4, 3721–3734, 10.3390/curroncol30040283. PMC10136780 37185396

[29] Fang Q. G. , Shi S. , and Li Z. N. , et al.Squamous Cell Carcinoma of the Buccal Mucosa: Analysis of Clinical Presentation, Outcome and Prognostic Factors, Molecular and Clinical Oncology. (2013) 1, no. 3, 531–534, 10.3892/mco.2013.86. PMC3915914 24649206

[30] Saghravanian N. , Ghazi N. , Meshkat Z. , and Mohtasham N. , Human Papillomavirus in Oral Leukoplakia, Verrucous Carcinoma, Squamous Cell Carcinoma, and Normal Mucous Membrane, Oman Medical Journal. (2015) 30, no. 6, 455–460, 10.5001/omj.2015.89. PMC4678447 26674929

[31] Alramadhan S. A. and Islam M. N. , Vesiculobullous Lesions of the Oral Cavity, Oral and Maxillofacial Surgery Clinics of North America. (2023) 35, no. 2, 203–217, 10.1016/j.coms.2022.10.006. 37019505

[32] Leuci S. , Ruoppo E. , Adamo D. , Calabria E. , and Mignogna M. D. , Oral Autoimmune Vesicobullous Diseases: Classification, Clinical Presentations, Molecular Mechanisms, Diagnostic Algorithms, and Management, Periodontology 2000. (2019) 80, no. 1, 77–88, 10.1111/prd.12263. 31090146

[33] Carra M. C. , Wasielewski C. , Špiljak B. , Sabalić-Schoener M. , Jungo S. , and Alajbeg I. , Management of Non-Plaque-Induced Gingival Conditions: A Systematic Review—Part 2: Inflammatory and Immune Conditions; Neoplasms; and Gingival Pigmentation, Journal of Clinical Periodontology. (2026) 0, 1–37, 10.1111/jcpe.70152. 42321019

[34] Davis G. , Hathway R. , Shipley D. , and Staines K. , The Management of *Pemphigus vulgaris* and Mucous Membrane Pemphigoid in a Joint Oral Medicine and Dermatology Clinic: A 5-Year Narrative Review, British Dental Journal. (2024) 236, no. 4, 311–316, 10.1038/s41415-024-7074-8. PMC10883883 38388610

[35] Bagan J. , Jiménez Y. , Murillo J. , and Bagan L. , Oral Mucous Membrane Pemphigoid: A Clinical Study of 100 Low-Risk Cases, Oral Diseases. (2018) 24, no. 1-2, 132–134, 10.1111/odi.12744. 29480624

[36] Di Zenzo G. , Carrozzo M. , and Chan L. S. , Urban Legend Series: Mucous Membrane Pemphigoid, Oral Diseases. (2014) 20, no. 1, 35–54, 10.1111/odi.12193. 24320968

[37] Esmaili N. , Mortazavi H. , and Noormohammadpour P. , et al. *Pemphigus vulgaris* and Infections: A Retrospective Study on 155 Patients, Autoimmune Diseases. (2013) 2013, 10.1155/2013/834295, 834295. PMC3697281 23844280

[38] Geng R. S. , Wilken B. , Sood S. , Sibbald R. G. , and Sibbald C. , Biomarkers in Pemphigus Vulgaris: A Systematic Review, Journal of Cutaneous Medicine and Surgery. (2024) 28, no. 5, 458–462, 10.1177/12034754241266136. PMC11528842 39075718

[39] Ohki M. and Kikuchi S. , Nasal, Oral, and Pharyngolaryngeal Manifestations of Pemphigus Vulgaris: Endoscopic Ororhinolaryngologic Examination, Ear, Nose & Throat Journal. (2017) 96, no. 3, 120–127, 10.1177/014556131709600311. 28346642

[40] Wang J. and Van der Waal I. , Disease Scoring Systems for Oral Lichen Planus; a Critical Appraisal, Medicina Oral Patología Oral y Cirugia Bucal. (2015) 20, e199–e204, 10.4317/medoral.20524. PMC4393983 25681372

[41] Warnakulasuriya S. , White, Red, and Mixed Lesions of Oral Mucosa: A Clinicopathologic Approach to Diagnosis, Periodontology 2000. (2019) 80, no. 1, 89–104, 10.1111/prd.12276. 31090142

[42] Alrashdan M. S. , Cirillo N. , and McCullough M. , Oral Lichen Planus: A Literature Review and Update, Archives of Dermatological Research. (2016) 308, no. 8, 539–551, 10.1007/s00403-016-1667-2. 27349424

[43] Budimir J. , Mihić L. L. , Situm M. , Bulat V. , Persić S. , and Tomljanović-Veselski M. , Oral Lesions in Patients With *Pemphigus vulgaris* and Bullous Pemphigoid, Acta Clinica Croatica. (2008) 47, no. 1, 8–13. 18714642

[44] Hammers C. M. and Stanley J. R. , Mechanisms of Disease: Pemphigus and Bullous Pemphigoid, Annual Review of Pathology: Mechanisms of Disease. (2016) 11, no. 1, 175–197, 10.1146/annurev-pathol-012615-044313. PMC5560122 26907530

[45] Reichart P. A. , Oral Manifestations in HIV Infection: Fungal and Bacterial Infections, Kaposi’s Sarcoma, Medical Microbiology and Immunology. (2003) 192, no. 3, 165–169, 10.1007/s00430-002-0175-5. 12684760

[46] Ramos-Gomez F. J. , Flaitz C. , Catapano P. , Murray P. , Milnes A. R. , and Dorenbaum A. , Classification, Diagnostic Criteria, and Treatment Recommendations for Orofacial Manifestations in HIV-Infected Pediatric Patients. Collaborative Workgroup on Oral Manifestations of Pediatric HIV Infection, Journal of Clinical Pediatric Dentistry. (1999) 23, no. 2, 85–96. 10204447

[47] Lamster I. B. , Grbic J. T. , Mitchell-Lewis D. A. , Begg M. D. , and Mitchell A. , New Concepts Regarding the Pathogenesis of Periodontal Disease in HIV Infection, Annals of Periodontology. (1998) 3, no. 1, 62–75, 10.1902/annals.1998.3.1.62. 9722691

[48] Holmstrup P. and Westergaard J. , HIV Infection and Periodontal Diseases, Periodontology 2000. (1998) 18, no. 1, 37–46, 10.1111/j.1600-0757.1998.tb00137.x. 10200711

[49] Portela M. B. , Cerqueira D. F. , de Paiva R. V. , Santos A. M. , Vargas T. R. , and Castro G. F. , Candida Spp. in Linear Gingival Erythema Lesions in Hiv-Infected Children: Reports of Six Cases, Revista Fluminense de Odontologia. (2018) 3, 42–46.

[50] Gunawan I. and Dewi T. S. , Oral Lichen Planus Lesion Mimicking Linear Gingival Erythema: A Case Report, Journal of Syiah Kuala Dentistry Society. (2017) 2, 40–45.

[51] Al Beesh F. A. , Martini N. , Suleiman S. , and Aljoujou A. , Oral Manifestations Associated With Neutropenia in Syrian Patients Diagnosed With Hematological Malignancies and Undergoing Chemotherapy: A Cross-Sectional Study, Medicine. (2024) 103, no. 2, 10.1097/MD.0000000000036780, e36780. PMC10783351 38215147

[52] Gates G. F. , Chronic Neutropenia Presenting With Oral Lesions, Oral Surgery, Oral Medicine, Oral Pathology. (1969) 27, no. 4, 563–567, 10.1016/0030-4220(69)90081-4.

[53] Gorlin R. J. and Chaudhry A. P. , The Oral Manifestations of Cyclic (Periodic) Neutropenia: Does Periadenitis Mucosa Necrotica Recurrens (Sutton) Exist? A Need for Revaluation, Archives of Dermatology. (1960) 82, no. 3, 344–348, 10.1001/archderm.1960.01580030038004. 13851317

[54] Scully C. , MacFadyen E. , and Campbell A. , Oral Manifestation in Cyclic Neutropenia, British Journal of Oral Surgery. (1982) 20, no. 2, 96–101, 10.1016/0007-117X(82)90015-4. 6954986

[55] da Fonseca M. A. and Fontes F. , Early Tooth Loss due to Cyclic Neutropenia: Long-Term Follow-Up of One Patient, Special Care in Dentistry. (2000) 20, no. 5, 187–190, 10.1111/j.1754-4505.2000.tb00017.x. 11203896

[56] Nakai Y. , Ishihara C. , Ogata S. , and Shimono T. , Oral Manifestations of Cyclic Neutropenia in a Japanese Child: Case Report With a 5-Year Follow-Up, Pediatric Dentistry. (2003) 25, 383–388. 13678105

[57] Birt D. , From L. , and Main J. , Oral Manifestations of Herpes Simplex Virus Infections, The Laryngoscope. (1977) 87, no. 6, 872–878, 10.1288/00005537-197706000-00004. 865205

[58] Mortazavi H. , Safi Y. , Baharvand M. , Rahmani S. , and Jafari S. , Peripheral Exophytic Oral Lesions: A Clinical Decision Tree, International Journal of Dentistry. (2017) 2017, 10.1155/2017/9193831, 9193831. PMC5516740 28757870

[59] Gawron K. , Łazarz-Bartyzel K. , Potempa J. , and Chomyszyn-Gajewska M. , Gingival Fibromatosis: Clinical, Molecular and Therapeutic Issues, Orphanet Journal of Rare Diseases. (2016) 11, no. 1, 1–4, 10.1186/s13023-016-0395-1. PMC4729029 26818898

[60] Brierley D. J. , Crane H. , and Hunter K. D. , Lumps and Bumps of the Gingiva: A Pathological Miscellany, Head and Neck Pathology. (2019) 13, no. 1, 103–113, 10.1007/s12105-019-01000-w. PMC6404662 30693455

[61] Chojnacka-Purpurowicz J. , Wygonowska E. , Placek W. , and Owczarczyk-Saczonek A. , Cyclosporine-Induced Gingival Overgrowth, Dermatologic Therapy. (2022) 35, no. 12, 10.1111/dth.15912, e15912. 36208445

[62] Sangle V. A. , Pooja V. K. , Holani A. , Shah N. , Chaudhary M. , and Khanapure S. , Reactive Hyperplastic Lesions of the Oral Cavity: A Retrospective Survey Study and Literature Review, Indian Journal of Dental Research. (2018) 29, no. 1, 61–66, 10.4103/ijdr.IJDR_599_16. 29442089

[63] Truschnegg A. , Pichelmayer M. , Acham S. , and Jakse N. , Nonsurgical Treatment of an Epulis by Photodynamic Therapy, Photodiagnosis and Photodynamic Therapy. (2016) 14, 1–3, 10.1016/j.pdpdt.2016.02.003. 26854720

[64] Lester S. R. , Cordell K. G. , Rosebush M. S. , Palaiologou A. A. , and Maney P. , Peripheral Giant Cell Granulomas: A Series of 279 Cases, Oral Surgery, Oral Medicine, Oral Pathology and Oral Radiology. (2014) 118, no. 4, 475–482, 10.1016/j.oooo.2014.06.004. 25240995

[65] Naderi N. J. , Eshghyar N. , and Esfehanian H. , Reactive Lesions of the Oral Cavity: A Retrospective Study on 2068 Cases, Journal of Dental Research. (2012) 9, 251. PMC3469888 23087727

[66] Vidyanath S. , Shameena P. M. , Johns D. A. , Shivashankar V. Y. , Sudha S. , and Varma S. , Reactive Hyperplasic Lesions of the Oral Cavity: A Survey of 295 Cases at a Tertiary Health Institution in Kerala, Journal of Oral and Maxillofacial Pathology. (2015) 19, no. 3, 330–334, 10.4103/0973-029X.174614. PMC4774286 26980961

[67] Lomeli Martinez S. M. , Carrillo Contreras N. G. , and Gómez Sandoval J. R. , et al.Oral Pyogenic Granuloma: A Narrative Review, International Journal of Molecular Sciences. (2023) 24, no. 23, 10.3390/ijms242316885, 16885. PMC10706684 38069207

[68] McNamara K. K. and Kalmar J. R. , Erythematous and Vascular Oral Mucosal Lesions: A Clinicopathologic Review of Red Entities, Head and Neck Pathology. (2019) 13, no. 1, 4–15, 10.1007/s12105-019-01002-8. PMC6405795 30693460

[69] Flaitz C. M. , Peripheral Giant Cell Granuloma: A Potentially Aggressive Lesion in Children, Pediatric Dentistry. (2000) 22, no. 3, 232–233. 10846736

[70] Nekouei A. , Eshghi A. , Jafarnejadi P. , and Enshaei Z. , A Review and Report of Peripheral Giant Cell Granuloma in a 4-Year-Old Child, Case Reports in Dentistry. (2016) 2016, 10.1155/2016/7536304, 7536304. PMC4925949 27403351

[71] Buchner A. and Hansen L. S. , The Histomorphologic Spectrum of Peripheral Ossifying Fibroma, Oral Surgery, Oral Medicine, Oral Pathology. (1987) 63, no. 4, 452–461, 10.1016/0030-4220(87)90258-1. 3472146

[72] Poon C. K. , Kwan P. C. , and Chao S. Y. , Giant Peripheral Ossifying Fibroma of the Maxilla: Report of a Case, Journal of Oral and Maxillofacial Surgery. (1995) 53, no. 6, 695–698, 10.1016/0278-2391(95)90174-4. 7776054

[73] Farquhar T. , MacLellan J. , Dyment H. , and Anderson R. D. , Peripheral Ossifying Fibroma: A Case Report, Journal of the Canadian Dental Association. (2008) 74, no. 9, 809–812. 19000465

[74] Epivatianos A. , Antoniades D. , and Zaraboukas T. , et al.Pyogenic Granuloma of the Oral Cavity: Comparative Study of Its Clinicopathological and Immunohistochemical Features, Pathology International. (2005) 55, no. 7, 391–397, 10.1111/j.1440-1827.2005.01843.x. 15982213

[75] Nikitakis N. G. , Oral Soft Tissue Lesions: A Guide to Differential Diagnosis Part I: Introduction and Changes in Color, Brazilian Journal of Oral Sciences. (2003) 2, 291–299.

[76] Reddy V. , Saxena S. , Saxena S. , and Reddy M. , Reactive Hyperplastic Lesions of the Oral Cavity: A Ten Year Observational Study on North Indian Population, Journal of Clinical and Experimental Dentistry. (2012) 4, e136–e140, 10.4317/jced.50670. PMC3917636 24558543

[77] Khalighi H. R. , Hamian M. , Abbas F. M. , and Farhadi S. , Simultaneous Existence of Giant Cell Fibroma and Squamous Papilloma in the Oral Cavity, Indian Journal of Medical Specialities. (2011) 2, no. 2, 153–156, 10.7713/ijms.2011.0038.

[78] Betz S. J. , HPV-Related Papillary Lesions of the Oral Mucosa: A Review, Head and Neck Pathology. (2019) 13, no. 1, 80–90, 10.1007/s12105-019-01003-7. PMC6405797 30693456

[79] Sugimoto K. J. , Shimada A. , and Sakurai H. , et al.Primary Gingival Diffuse Large B-Cell Lymphoma: A Case Report and a Review of the Literature, International Journal of Clinical and Experimental Pathology. (2013) 7, 418. PMC3885500 24427366

[80] Bagan J. V. , Carbonell F. , and Gómez M. J. , et al.Extra-Nodal B-Cell Non-Hodgkin’s Lymphomas of the Head and Neck: A Study of 68 Cases, American Journal of Otolaryngology. (2015) 36, no. 1, 57–62, 10.1016/j.amjoto.2014.10.008. 25456517

[81] Silva T. D. , Ferreira C. B. , Leite G. B. , de Menezes Pontes J. R. , and Antunes H. S. , Oral Manifestations of Lymphoma: A Systematic Review, Ecancermedicalscience. (2016) 10, 10.3332/ecancer.2016.665, 665. PMC4990057 27594910

[82] Ison J. and Clark A. , Pigmented Lesions of the Oral Cavity, Oral and Maxillofacial Surgery Clinics of North America. (2023) 35, no. 2, 153–158, 10.1016/j.coms.2022.10.008. 37019502

[83] Wu J. , Fantasia J. E. , and Kaplan R. , Oral Manifestations of Acute Myelomonocytic Leukemia: A Case Report and Review of the Classification of Leukemias, Journal of Periodontology. (2002) 73, no. 6, 664–668, 10.1902/jop.2002.73.6.664. 12083541

[84] Allon I. , Pessing A. , Kaplan I. , Allon D. M. , and Hirshberg A. , Metastatic Tumors to the Gingiva and the Presence of Teeth as a Contributing Factor: A Literature Analysis, Journal of Periodontology. (2014) 85, no. 1, 132–139, 10.1902/jop.2013.130118. 23646853

[85] Comarmond C. and Cacoub P. , Granulomatosis With Polyangiitis (Wegener): Clinical Aspects and Treatment, Autoimmunity Reviews. (2014) 13, no. 11, 1121–1125, 10.1016/j.autrev.2014.08.017. 25149391

[86] Stewart C. , Cohen D. , and Bhattacharyya I. , et al.Oral Manifestations of Wegener’s Granulomatosis: A Report of Three Cases and a Literature Review, The Journal of the American Dental Association. (2007) 138, no. 3, 338–348, 10.14219/jada.archive.2007.0166. 17332039

[87] Batal H. , Chou L. L. , and Cottrell D. A. , Sarcoidosis: Medical and Dental Implications, Oral Surgery, Oral Medicine, Oral Pathology, Oral Radiology, and Endodontology. (1999) 88, no. 4, 386–390, 10.1016/S1079-2104(99)70049-1. 10519742

[88] Blinder D. , Yahatom R. , and Taicher S. , Oral Manifestations of Sarcoidosis, Oral Surgery, Oral Medicine, Oral Pathology, Oral Radiology, and Endodontology. (1997) 83, no. 4, 458–461, 10.1016/S1079-2104(97)90145-1. 9127377

[89] Chisholm D. M. , Lyell A. , Haroon T. S. , Mason D. K. , and Beeley J. A. , Salivary Gland Function in Sarcoidosis: Report of a Case, Oral Surgery, Oral Medicine, Oral Pathology. (1971) 31, no. 6, 766–771, 10.1016/0030-4220(71)90131-9. 5280458

[90] Bajkovec L. , Mrzljak A. , Likic R. , and Alajbeg I. , Drug-Induced Gingival Overgrowth in Cardiovascular Patients, World Journal of Cardiology. (2021) 13, no. 4, 68–75, 10.4330/wjc.v13.i4.68. PMC8069521 33968305

[91] Seymour R. A. , Ellis J. S. , and Thomason J. M. , Risk Factors for Drug-Induced Gingival Overgrowth, Journal of Clinical Periodontology. (2000) 27, no. 4, 217–223, 10.1034/j.1600-051x.2000.027004217.x. 10783833

[92] Livada R. and Shiloah J. , Calcium Channel Blocker-Induced Gingival Enlargement, Journal of Human Hypertension. (2014) 28, no. 1, 10–14, 10.1038/jhh.2013.47. 23739159

[93] Fardal Ø. and Lygre H. , Management of Periodontal Disease in Patients Using Calcium Channel Blockers–Gingival Overgrowth, Prescribed Medications, Treatment Responses and Added Treatment Costs, Journal of Clinical Periodontology. (2015) 42, no. 7, 640–646, 10.1111/jcpe.12426. 26076712

[94] Schroeder H. E. and Shanley D. , Formation and Inhibition of Dental Calculus, Journal of Periodontology. (1969) 40, no. 11, 643–646, 10.1902/jop.1969.40.11.643. 5260623

[95] Mignogna M. D. , Fortuna G. , and Leuci S. , et al.Frictional Keratoses on the Facial Attached Gingiva Are Rare Clinical Findings and Do Not Belong to the Category of Leukoplakia, Journal of Oral and Maxillofacial Surgery. (2011) 69, no. 5, 1367–1374, 10.1016/j.joms.2010.05.087. 21216078

[96] Nagao T. , Warnakulasuriya S. , and Hasegawa S. , et al.Elucidating Risk Factors for Oral Leukoplakia Affecting Gingivae in Japanese Subjects, Translational Research in Oral Oncology. (2016) 1, 10.1177/2057178X16654704, 2057178X16654704.

[97] Kumari P. , Debta P. , and Dixit A. , Oral Potentially Malignant Disorders: Etiology, Pathogenesis, and Transformation Into Oral Cancer, Frontiers in Pharmacology. (2022) 13, 10.3389/fphar.2022.825266, 825266. PMC9065478 35517828

[98] Mortazavi H. , Baharvand M. , and Mehdipour M. , Oral Potentially Malignant Disorders: An Overview of More Than 20 Entities, Journal of Dental Research, Dental Clinics, Dental Prospects. (2014) 8, 6. 10.5681/joddd.2014.002 PMC4091702 25024833

[99] Parlatescu I. , Gheorghe C. , Coculescu E. , and Tovaru S. , Oral Leukoplakia–An Update, Maedica. (2014) 9, 88. PMC4268300 25553134

[100] Kato M. G. , Baek C. H. , and Chaturvedi P. , et al.Update on Oral and Oropharyngeal Cancer Staging-International Perspectives, World Journal of Otorhinolaryngology –Head and Neck Surgery. (2020) 6, no. 1, 66–75, 10.1016/j.wjorl.2019.06.001. PMC7221211 32426706

[101] Zargaran M. , Eshghyar N. , Vaziri P. B. , and Mortazavi H. , Immunohistochemical Evaluation of Type IV Collagen and Laminin-332 γ2 Chain Expression in Well-Differentiated Oral Squamous Cell Carcinoma and Oral Verrucous Carcinoma: A New Recommended Cut-Off, Journal of Oral Pathology & Medicine. (2011) 40, no. 2, 167–173, 10.1111/j.1600-0714.2010.00983.x. 21158930

[102] Alkan A. , Bulut E. , Gunhan O. , and Ozden B. , Oral Verrucous Carcinoma: A Study of 12 Cases, European Journal of Dentistry. (2019) 04, no. 2, 202–207, 10.1055/s-0039-1697831. PMC2853822 20396454

[103] Candau-Alvarez A. , Dean-Ferrer A. , and Alamillos-Granados F. J. , et al.Verrucous Carcinoma of the Oral Mucosa: An Epidemiological and Follow-up Study of Patients Treated With Surgery in 5 last Years, Medicina Oral Patología Oral y Cirugia Bucal. (2014) 19, e506–e511, 10.4317/medoral.19683. PMC4192576 24880446

[104] Capella D. L. , Gonçalves J. M. , Abrantes A. A. , Grando L. J. , and Daniel F. I. , Proliferative Verrucous Leukoplakia: Diagnosis, Management and Current Advances, Brazilian Journal of Otorhinolaryngology. (2017) 83, no. 5, 585–593, 10.1016/j.bjorl.2016.12.005. PMC9444738 28209441

[105] Munde A. and Karle R. , Proliferative Verrucous Leukoplakia: An Update, Journal of Cancer Research and Therapeutics. (2016) 12, no. 2, 469–473, 10.4103/0973-1482.151443. 27461595

[106] Martorell-Calatayud A. , Botella-Estrada R. , Bagán-Sebastián J. V. , Sanmartín-Jiménez O. , and Guillén-Barona C. , Oral Leukoplakia: Clinical, Histopathologic, and Molecular Features and Therapeutic Approach, Actas Dermo-Sifiliográficas. (2009) 100, no. 8, 669–684, 10.1016/S1578-2190(09)70149-0. 19775545

[107] Rosebush M. S. , Briody A. N. , and Cordell K. G. , Black and Brown: Non-Neoplastic Pigmentation of the Oral Mucosa, Head and Neck Pathology. (2019) 13, no. 1, 47–55, 10.1007/s12105-018-0980-9. PMC6405786 30671761

[108] Feller L. , Masilana A. , Khammissa R. A. , Altini M. , Jadwat Y. , and Lemmer J. , Melanin: The Biophysiology of Oral Melanocytes and Physiological Oral Pigmentation, Head & Face Medicine. (2014) 10, no. 1, 1–7, 10.1186/1746-160X-10-8. PMC3994327 24661309

[109] Müller S. , Melanin-Associated Pigmented Lesions of the Oral Mucosa: Presentation, Differential Diagnosis, and Treatment, Dermatologic Therapy. (2010) 23, no. 3, 220–229, 10.1111/j.1529-8019.2010.01319.x. 20597941

[110] Amir E. , Gorsky M. , Buchner A. , Sarnat H. , and Gat H. , Physiologic Pigmentation of the Oral Mucosa in Israeli Children, Oral Surgery, Oral Medicine, Oral Pathology. (1991) 71, no. 3, 396–398, 10.1016/0030-4220(91)90325-7. 2011372

[111] Parizi J. L. and Nai G. A. , Amalgam Tattoo: A Cause of Sinusitis?, Journal of Applied Oral Science. (2010) 18, no. 1, 100–104, 10.1590/S1678-77572010000100016. PMC5349031 20379688

[112] Buchner A. and Hansen L. S. , Amalgam Pigmentation (Amalgam Tattoo) of the Oral Mucosa: A Clinicopathologic Study of 268 Cases, Oral Surgery, Oral Medicine, Oral Pathology. (1980) 49, no. 2, 139–147, 10.1016/0030-4220(80)90306-0. 6928285

[113] Martin J. M. , Nagore E. , and Cremades A. , et al.An Amalgam Tattoo on the Oral Mucosa Related to a Dental Prosthesis, Journal of the European Academy of Dermatology and Venereology. (2005) 19, no. 1, 90–92, 10.1111/j.1468-3083.2004.01071.x. 15649199

[114] Sortino-Rachou A. M. , de Camargo Cancela M. , Voti L. , and Curado M. P. , Primary Oral Melanoma: Population-Based Incidence, Oral Oncology. (2009) 45, no. 3, 254–258, 10.1016/j.oraloncology.2008.04.015. 18675580

[115] Pathak S. and Zito P. M. , Clinical Guidelines for the Staging, Diagnosis, and Management of Cutaneous Malignant Melanoma, 2025, StatPearls.

[116] Azarisamani A. , Petrisor D. , Wright J. , and Ghali G. E. , Metastatic Melanotic Neuroectodermal Tumor of Infancy: Report of a Case and Review of the Literature, Journal of Oral and Maxillofacial Surgery. (2016) 74, no. 12, 2431–2440, 10.1016/j.joms.2016.05.028. 27393580

[117] Souza P. R. , Dupont L. , Mosena G. , Dantas M. L. , and Bulcão L. A. , Variations of Oral Anatomy and Common Oral Lesions, Anais Brasileiros De Dermatologia. (2024) 99, no. 1, 3–18, 10.1016/j.abd.2023.06.001. PMC10964358 37722995

[118] Kaugars G. E. , Heise A. P. , Riley W. T. , Abbey L. M. , and Svirsky J. A. , Oral Melanotic Macules: A Review of 353 Cases, Oral Surgery, Oral Medicine, Oral Pathology. (1993) 76, no. 1, 59–61, 10.1016/0030-4220(93)90295-F. 8351123

[119] Alawi F. , Pigmented Lesions of the Oral Cavity, Dental Clinics of North America. (2013) 57, no. 4, 699–710, 10.1016/j.cden.2013.07.006. PMC3775277 24034073

[120] Muñoz-Corcuera M. , Esparza-Gómez G. , González-Moles M. A. , and Bascones-Martínez A. , Oral Ulcers: Clinical Aspects. A Tool for Dermatologists, Clinical and Experimental Dermatology. (2009) 34, no. 4, 456–461, 10.1111/j.1365-2230.2009.03219.x. 19522982

[121] Harris P. , Bissonnette C. , Tabet P. , and Wittmer R. , Common White Lesions of the Oral Cavity: Review of Clinical Presentations and Management, Canadian Family Physician. (2025) 71, no. 1, 19–25, 10.46747/cfp.710119. PMC11753272 39843200

[122] Gondak R. O. , da Silva-Jorge R. , Jorge J. , Lopes M. A. , and Vargas P. A. , Oral Pigmented Lesions: Clinicopathologic Features and Review of the Literature, Medicina Oral Patología Oral y Cirugia Bucal. (2012) 17, e919–e924, 10.4317/medoral.17679. PMC3505710 22549672

[123] Zahid E. , Bhatti O. , Zahid M. A. , and Stubbs M. , Overview of Common Oral Lesions, Malaysian Family Physician. (2022) 17, 10.51866/mfp1985, 9. PMC9809440 36606178

[124] Krahl D. , Altenburg A. , and Zouboulis C. C. , Reactive Hyperplasias, Precancerous and Malignant Lesions of the Oral Mucosa, JDDG: Journal der Deutschen Dermatologischen Gesellschaft. (2008) 6, no. 3, 217–232, 10.1111/j.1610-0387.2007.06625.x. 18315622

[125] Kerr A. R. and Lodi G. , Management of Oral Potentially Malignant Disorders, Oral Diseases. (2021) 27, no. 8, 2008–2025, 10.1111/odi.13980. 34324758

